# Ag_2_O on ZrO_2_ as a Recyclable Catalyst for Multicomponent Synthesis of Indenopyrimidine Derivatives

**DOI:** 10.3390/molecules23071648

**Published:** 2018-07-05

**Authors:** Sandeep V. H. S. Bhaskaruni, Suresh Maddila, Werner E. van Zyl, Sreekantha B. Jonnalagadda

**Affiliations:** School of Chemistry & Physics, University of KwaZulu-Natal, Westville Campus, Chiltern Hills, Durban 4000, South Africa; Saisandeep93@gmail.com (S.V.H.S.B.); sureshmskt@gmail.com (S.M.); vanzylw@ukzn.ac.za (W.E.v.Z.)

**Keywords:** indenopyrimidines, no column chromatography, Ag_2_O/ZrO_2_, heterogeneous catalysis, mixed oxides, green chemistry

## Abstract

We describe the synthesis of silver loaded on zirconia and its use as an efficient catalyst for a one-pot three-component reaction to synthesize 11 indenopyrimidine derivatives, of which 7 are new compounds. The procedure involves substituted benzaldehydes, indane-1,3-dione, and guanidinium hydrochloride, with ethanol as solvent. The proposed green protocol at room temperature is simple and efficient, giving excellent yields (90–96%) in short reaction times (<30 min). The protocol works well according to the green chemistry principles with respect to high atom economy, no need for column separation, and reusability of the catalyst, which are attractive features. XRD, TEM, SEM, and BET analysis were used to characterize the catalyst materials.

## 1. Introduction

Multicomponent reactions (MCRs) are cherished protocols in organic synthesis due to their vast potential in the fields of medicinal, pharmaceutical, and agro-chemistry [1]. MCRs are different from the other classical synthesis approaches due to their facile synthesis protocols, high atom efficiency, high yields, and minimal by-product formation [2]. Further, the efficacy of MCRs when compared with multistep synthetic protocols lies in the selective formation of several bonds in a single reaction flask with highly desirable yield over a short interval of time [3].

Currently, heterogeneous catalysts play a key role in organic synthesis because they meet most of the goals of green and sustainable chemistry [4]. Researchers have made remarkable improvements in the design of well-defined catalyst materials. Innovative methods have allowed the coherent design and preparation of very active and selective catalyst materials by governing the structure and composition of the active particles [5]. Furthermore, heterogeneous catalysts offer many benefits in synthetic transformations such as outstanding chemical and thermal stability, noncorrosiveness, nonflammability, eco-friendliness, nontoxicity, ease of separation, reusability, and commercial availability [6].

In recent years, zirconium oxide (ZrO_2_) have been effusively revealed as a catalyst or a supported catalyst in various potential chemical applications due to its extensive properties such as acidic and basic sites [7], high stability in the presence of redox conditions, active phase support, and chemical consistency related to other supports like alumina and silica. Further, ZrO_2_ is low cost, stable, nonhazardous, reusable, and readily available [8]. Silver salts are widely used as catalysts and their efficacy has been demonstrated in terms of being cost-effective, non-toxic, simple to handle, and appropriate for usage as a one-electron redox system [9]. However, their use in large amounts is unwarranted, but if used in a green-manner as a recyclable catalyst, it has many advantages. Hence, the use of silver-loaded zirconia catalysts is a desirable choice for eco-friendly synthesis.

Among the known heterocyclic compounds, N-heterocyclic scaffolds are very important in medicinal chemistry [10]. In the N-heterocyclic class of scaffolds, pyrimidine and its derivatives play a prominent role [11]. Being the main constituents of several natural products, these pyrimidines represent a significant class of molecules which have been of increased interest in recent years owing to their valuable pharmacological and biological applications in antimicrobial [12], antifungal [13], anti-tumor [14], anti-HIV [15], anti-tubercular [16], anti-inflammatory [17], and antimalarial [18] activities. Moreover, aryl-indenopyrimidines have been used as adenosine A2A receptor antagonists [19], which are useful, and many reports have dealt with the preparation of different types of pyrimidine derivatives via multicomponent reactions. Owing to their economic viability and scientific importance, methodologies have been described for the synthesis of various types of substituted pyrimidines. Diverse catalytic systems such as NaOH, references [20,21,22] sodium methoxide [23], 1-sulfonic acid-3-methyl imidazolium ferric chloride/NaY [24], α-Fe_2_O_3_-MCM-41-P [25], and uranyl acetate/succinimide sulfonic acid [26] have been used in the synthesis of pyrimidine derivatives. However, these protocols invariably demand expensive reagents, high energy input, and lengthy reaction times. Other limitations are lower yields and the need for solvents and column separations. There is, thus, a demand for a greener preparation method for indenopyrimidines. We found no literature reports to date using metal complexes or metal oxides as catalysts in their synthesis. Although silver-loaded ZrO_2_ have been reported as a catalyst for varied reactions [27,28], no protocols have been described for the synthesis of indenopyrimidines via MCRs.

We recently reported several green synthetic approaches of various medicinally interesting heterocyclic scaffolds [29,30,31,32,33,34,35,36,37]. Encouraged by those favorable results with different substituted heterocyclic scaffolds, here we describe the synthesis and characterization of silver loaded on zirconia and its efficiency in the one-pot synthesis of functionalized indenopyrimidines via a three-component reaction at room temperature (RT).

## 2. Experimental Section

### 2.1. Catalyst Preparation

Supported catalysts with different wt % (weight percentage) of silver-loaded zirconia (1, 2.5, and 5.0 wt %) were synthesized following a wet impregnation process [38,39]. The solid heterogeneous catalyst was prepared by combining zirconia (ZrO_2_, 3 g, Alfa Aesar, Ward Hill, MA, USA) and a suitable quantity of silver chloride (AgCl_2_, Alfa Aesar) dissolved in distillated water (60 mL). The mixture was stirred at room temperature (RT) for 5–7 h. After this time, the ensuing slurry was filtered under vacuum. It was dried in an oven at 120–130 °C for 6 h and calcined in the presence of air at 450 °C for 5 h to afford the (1.0, 2.5, and 5.0 wt %) silver on zirconia.

### 2.2. General Procedure for the Synthesis of Indenopyrimidine Derivatives

**Scheme 1:**
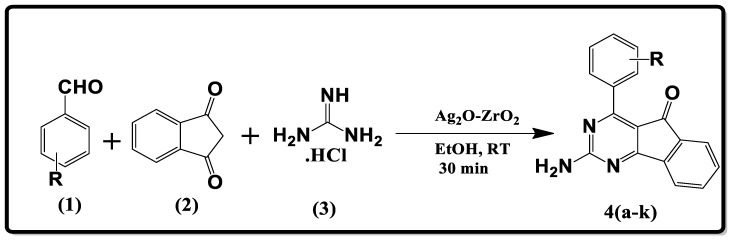
Synthesis of novel indenopyrimidine derivatives. RT = room temperature.

In a typical reaction, an equimolar mixture of aldehydes (1 mmol), indane-1,3-dione (1 mmol), and guanidinium hydrochloride (1 mmol) were dissolved in ethanol (5 mL) at RT, followed by the addition of Ag_2_O–ZrO_2_ (60 mg) as a catalyst. The reaction mixture was held at RT for 30 min under stirring (Scheme 1), while the reaction progress was monitored by TLC at regular time intervals. When the reaction was complete, the mixture was filtered, and ethyl acetate was used to extract the filtrate which was then evaporated under reduced pressure to obtain the crude product and washed with ethanol. The reaction product was recrystallized in ethanol to obtain the pure target compound. The yields reported are the weighed values after recrystallization and isolation. The products were further characterized by ^1^H-NMR, ^13^C-NMR, HRMS, and FT-IR analysis. The related details and spectra are given in the Supplementary Information File (SI-II & III).

*2-Amino-4-(2-methoxyphenyl)-5H-indeno[1,2–d]-pyrimidine-5-one* (**4a**). ^1^H-NMR (DMSO-*d*_6_, 400 MHz) δ = 3.83 (s, 3H, -OCH_3_), 7.09 (d, *J* = 7.6 Hz, 1H, Ar-H), 7.16 (d, *J* = 8.16 Hz, 1H, Ar-H), 7.61 (t, *J* = 1.2 Hz, 1H, Ar-H), 7.92–7.95 (m, 4H, Ar-H), 8.22 (s, 2H, -NH_2_), 8.77–8.79 (m, 1H, Ar-H). ^13^C-NMR δ = 56.03, 111.56, 120.10, 121.00, 122.94, 122.98, 128.16, 133.09, 135.69, 135.78, 135.86, 138.96, 139.32, 141.79, 160.05, 188.50, 189.61; FT-IR: 3269.10 (-NH), 2957.02 (-CH), 1710 (-C=O), 1593.28 (-C=C), 1483.73 (-C=N), 733.81 (-CH).

*2-Amino-4-(4-methoxyphenyl)-5H-indeno[1,2-d]-pyrimidine-5-one* (**4b**). ^1^H-NMR (DMSO-*d*_6_, 400 MHz) δ = 3.89 (s, 3H, -OCH_3_), 7.13 (d, *J* = 8.92 Hz, 2H, Ar-H), 7.80 (s, 2H, -NH_2_), 7.71 (s, 2H, NH_2_), 7.93–7.95 (m, 4H, Ar-H), 8.60 (d, *J* = 8.92 Hz, 2H, Ar-H). ^13^C-NMR δ = 55.72, 114.49, 122.86, 125.86, 126.28, 135.54, 135.69, 136.98, 139.27, 141.69, 145.72, 163.77, 188.96, 189.80; FT-IR: 3010.13 (-NH), 2941.01 (-CH), 1712.14 (-C=O), 1673.42 (-C=C), 1468.81 (-C=N), 771.95 (-CH); HRMS of [C_18_H_13_N_3_O_2_ + H]^+^ (*m*/*z*): 304.0766; Calcd: 304.0763.

*2-Amino-4-(2,3-dimethoxyphenyl)-5H-indeno[1,2-d]-pyrimidine-5-one* (**4c**). ^1^H-NMR (DMSO-*d*_6_, 400 MHz) δ = 3.91 (s, 6H, -OCH_3_), 7.21 (t, *J* = 8.08 Hz, 1H, Ar-H), 7.32–7.34 (m, 1H, Ar-H), 7.94–8.14 (m, 4H, Ar-H), 8.36 (s, 2H, -NH_2_), 8.38 (t, *J* = 1.04 Hz, 1H, Ar-H). ^13^C-NMR δ = 55.95, 61.49, 118.22, 123.05, 123.10, 123.61, 124.23, 126.21, 129.53, 136.02, 139.00, 139.42, 141.89, 150.23, 152.19; FT-IR: 3087.99 (-NH), 2939.24 (-CH), 1711.98 (-C=O), 1673.55 (-C=C), 1469.79 (-C=N), 771.94 (-CH); HRMS of [C_19_H_15_N_3_O_3_ + H]^+^ (*m*/*z*): 334.0992; Calcd: 334.1008.

*2-Amino-4-(2,5-dimethoxyphenyl)-5H-indeno[1,2-d]-pyrimidine-5-one* (**4d**). ^1^H-NMR (DMSO-*d*_6_, 400 MHz) δ = 3.82 (s, 3H, -OCH_3_), 3.89 (s, 3H, -OCH_3_), 7.11 (d, *J* = 9.12 Hz, 1H, Ar-H), 7.21 (d, *J* = 3.12 Hz, 1H, Ar-H), 7.93–7.97 (m, 4H, Ar-H), 8.23 (s, 2H, -NH_2_), 8.64 (d, *J* = 3.2 Hz, 1H, ArH). ^13^C-NMR δ = 55.51, 56.38, 112.72, 116.49, 121.32, 123.07, 128.11, 135.75, 135.89, 138.84, 139.29, 141.87, 152.29, 154.92, 188.82, 189.64, 206.58; FT-IR: 3087.87 (-NH), 2950.12 (-CH), 1746.67 (-C=O), 1683.90 (-C=C), 1588.60 (-C=N), 780.18 (-CH); HRMS of [C_19_H_15_N_3_O_3_ + H]^+^ (*m*/*z*): 334.0434; Calcd: 334.0441.

*2-Amino-4-(2-bromophenyl)-5H-indeno[1,2-d]-pyrimidine-5-one* (**4e**). ^1^H-NMR (DMSO-*d*_6_, 400 MHz) δ = 7.52–7.59 (m, 2H, NH_2_), 7.89–7.98 (m, 7H, Ar-H), 8.00 (s, 1H, Ar-H). ^13^C-NMR δ = 122.83, 122.91, 124.36, 130.67, 132.12, 132.32, 135.70, 135.92, 140.42, 141.20, 141.35, 141.67, 142.03, 142.98, 197.19, 198.68; FT-IR: 3089.08 (-NH), 2941.43 (-CH), 1745.65 (-C=O), 1673.02 (-C=C), 1564.46 (-C=N), 771.47 (-CH), 619.03 (-C-Br); HRMS of [C_17_H_10_BrN_3_O + H]^+^ (*m*/*z*): 353.1042; Calcd: 353.1042.

*2-Amino-4-(2-fluorophenyl)-5H-indeno[1,2-d]-pyrimidine-5-one* (**4f**). ^1^H-NMR (DMSO-*d*_6_, 400 MHz) δ =7.39 (s, 1H, NH_2_), 7.68 (t, *J* = 1.08 Hz, 2H, Ar-H), 7.92–7.99 (m, 6H, Ar-H). ^13^C-NMR δ = 115.65, 115.86, 123.24, 124.49, 124.52, 133.07, 136.07, 136.21, 139.60, 141.99, 160.66, 188.07, 188.85, 197.56, 198.69; FT-IR: 3275.94 (-NH), 2956.87 (-CH), 1740.56 (-C=O), 1620.19 (-C=C), 1485.01 (-C=N), 1008.10 (-C-F), 757.33 (-CH); HRMS of [C_17_H_10_BrN_3_O + H]^+^ (*m*/*z*): 292.0732; Calcd: 292.0732.

*2-Amino-4-(3,4-dimethoxyphenyl)-5H-indeno[1,2-d]-pyrimidine-5-one* (**4g**). ^1^H-NMR (DMSO-*d*_6_, 400 MHz) δ = 3.90 (d, *J* = 3.84 Hz, 6H, -OCH_3_), 7.16 (d, *J* = 8.52 Hz, 1H, Ar-H), 7.80 (s, 1H, NH_2_), 7.92–7.97 (m, 4H, Ar-H), 8.02–8.04 (m, 1H, Ar-H), 8.68 (d, *J* = 1.92 Hz, 1H, ArH). ^13^C-NMR δ = 55.18, 111.53, 115.74, 122.78, 122.91, 126.09, 131.08, 135.56, 135.69, 139.22, 146.43, 148.31, 153.91, 189.85; FT-IR: 3088.10 (-NH), 2942.06 (-CH), 1712.66 (-C=O), 1673.31 (-C=C), 1468.88 (-C=N), 772.61 (-CH), 801.96 (-CH).

*2-Amino-4-(3-hydroxyphenyl)-5H-indeno[1,2-d]-pyrimidine-5-one* (**4h**). ^1^H-NMR (DMSO-*d*_6_, 400 MHz) δ = 7.01–7.04 (m, 1H, Ar-H), 7.35 (t, *J* = 7.88 Hz, 1H, Ar-H), 7.71 (s, 2H, NH_2_), 7.82 (d, *J* = 7.72 Hz, 1H, Ar-H), 7.92–7.96 (m, 5H, Ar-H), 9.86 (s, 1H, -OH). ^13^C-NMR δ = 119.61, 120.67, 123.00, 123.04, 125.46, 128.94, 129.69, 133.84, 135.77, 135.93, 139.33, 141.89, 145.82, 157.33, 188.45, 189.43; FT-IR: 3241.25 (-OH), 3088.13 (-NH), 1745.77 (-C=O), 1665.42 (-C=C), 1484.83 (-C=N), 780.62 (-CH); HRMS of [C_17_H_11_N_3_O_2_ + H]^+^ (*m*/*z*): 290.0732; Calcd: 290.0732.

*2-Amino-4-(4-bromophenyl)-5H-indeno[1,2-d]-pyrimidine-5-one* (**4i**). ^1^H-NMR (DMSO-*d*_6_, 400 MHz) δ = 7.77 (d, *J* = 8.52 Hz, 2H, Ar-H), 7.81 (s, *J* = 8.56 Hz, 2H, -NH_2_), 7.95–7.99 (m, 4H, Ar-H), 8.41 (d, *J* = 8.52 Hz, 2H, Ar-H). ^13^C-NMR δ = 103.27, 109.58, 110.67, 112.60, 116.48, 136.51, 143.42, 151.63, 151.95, 152.67, 167.34, 194.19; FT-IR: 3087.96 (-NH), 1724.92 (-C=O), 1685.17 (-C=C), 1485.31 (-C=N), 828.42 (-CH), 606.46 (-C-Br).

*2-Amino-4-(4-chlorophenyl)-5H-indeno[1,2-d]-pyrimidine-5-one* (**4j**). ^1^H-NMR (DMSO-*d*_6_, 400 MHz) δ = 7.36 (d, *J* = 8.52 Hz, 2H, -NH_2_), 7.64 (d, *J* = 8.56 Hz, 1H, Ar-H), 7.85–7.97 (m, 6H, Ar-H), 7.82 (d, *J* = 7.72 Hz, 1H, Ar-H), 8.51 (d, *J* = 8.6 Hz, 1H, Ar-H). ^13^C-NMR δ = 122.57, 122.79, 123.13, 127.74, 127.82, 128.85, 130.79, 135.36, 135.59, 137.85, 141.51, 143.73, 197.94, 198.14, 199.07; FT-IR: 3089.02 (-NH), 1712.07 (-C=O), 1673.33 (-C=C), 1469.06 (-C=N), 802.02 (-CH), 702.10 (-C-Cl); HRMS of [C_17_H_10_ClN_3_O + H]^+^ (*m*/*z*): 308.1139; Calcd: 308.114.

*2-Amino-4-(4-ethylphenyl)-5H-indeno[1,2-d]-pyrimidine-5-one* (**4k**). ^1^H-NMR (DMSO-*d*_6_, 400 MHz) δ = 1.22 (t, *J* = 7.56 Hz, 3H, -CH_3_), 2.68–2.79 (m, 2H, -CH_2_), 7.42 (d, *J* = 8.2 Hz, 2H, Ar-H), 7.83 (s, 2H, -NH_2_), 7.93–7.96 (m, 4H, Ar-H), 8.46 (d, *J* = 8.24 Hz, 2H, Ar-H). ^13^C-NMR δ = 15.03, 28.42, 30.63, 122.56, 122.98, 123.04, 128.30, 130.48, 134.22, 135.57, 135.75, 135.90, 139.39, 141.86, 142.99, 145.64, 150.32, 188.69, 189.56, 206.47; FT-IR: 3088.40 (-NH), 2941.60 (-CH), 1712.33 (-C=O), 1673.31 (-C=C), 1469.87 (-C=N), 802.02 (-CH).

## 3. Results and Discussion

### 3.1. Crystallinity by Powder XRD (PXRD) Studies

**Figure 1:**
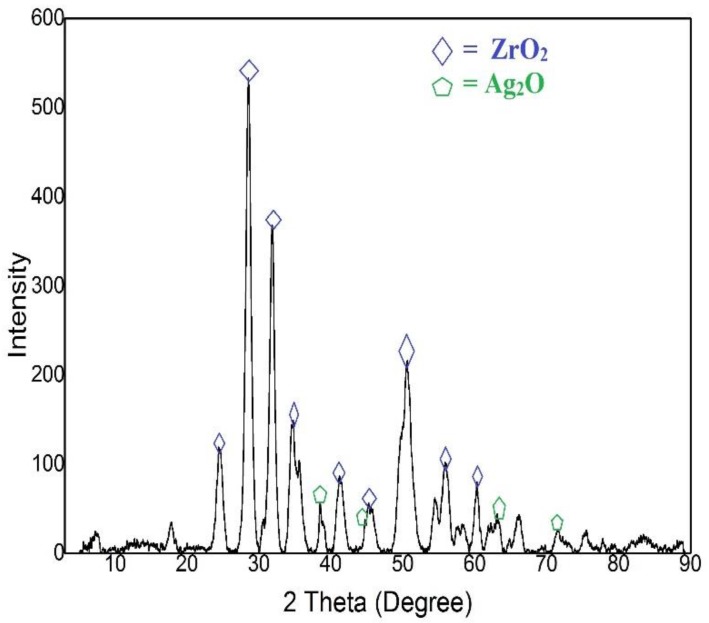
Powder X-ray diffractogram of 2.5% Ag_2_O–ZrO_2_ catalyst.

Figure 1 illustrates the crystalline phases of calcined silver oxide–zirconia catalyst material. The PXRD patterns of the catalytic sample display the major 2θ peak values at 24.2°, 28.2°, 31.3°, 35.4°, 40.5°, 45.0°, 50.3°, 55.4°, and 60.1° corresponding to zirconia; the peak values were correlated with the international standard file (JCPDS file no. 37-1484). In addition, the catalyst material revealed diffraction patterns at 2θ angles of 38.1°, 44.3°, 64.4°, and 77.4° corresponding to the Ag_2_O (JCPDS file no 72-2108). The peaks recognized in the diffractogram indicate the polycrystalline nature of the catalyst materials. Further, the average crystallite size of the catalyst was measured by the Debye–Scherrer formula using the strongest maximum-intensity diffraction peak, about 9.2 nm for 2.5% Ag_2_O/ZrO_2_.

### 3.2. TEM Analysis

**Figure 2:**
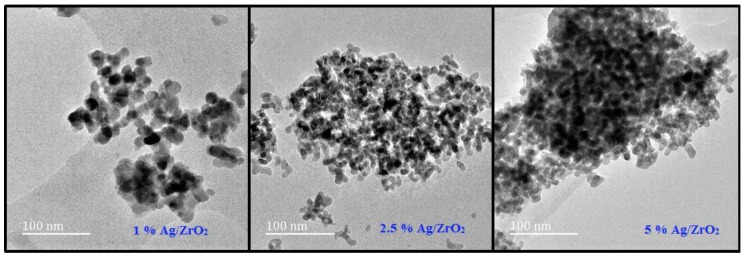
TEM micrographs of 1%, 2.5%, and 5% Ag_2_O–ZrO_2_ catalyst.

The transition electron microscopy images (Figure 2) provide the morphology of prepared Ag_2_O/ZrO_2_ of different wt %. In this image, the silver oxide particle dimensions are mostly in the range between 10 and 16 nm, with black spherical shapes and seemingly well distributed. Further, zirconia is revealed as white globular-shaped particles. It is clearly observed that 1% has a lesser dispersion of Ag, whereas 5% has more Ag agglomerated on the surface of Zr, which fails to provide more active sites to facilitate the reaction. Changes in the morphology of the recovered catalyst after reaction were marginal.

### 3.3. SEM Analysis

**Figure 3:**
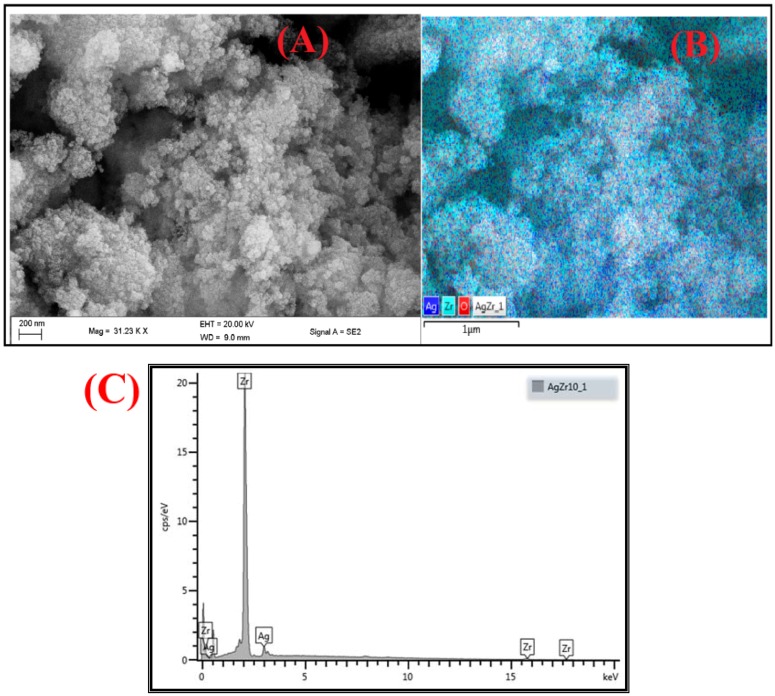
(**A**) SEM micrograph; (**B**) SEM mapping; (**C**) EDS spectra of 2.5% Ag_2_O–ZrO_2_ catalyst.

The morphology and size dispersion of the catalyst material were determined with SEM analysis. In Figure 3, a large number of white and irregular shapes are observed for 2.5% Ag_2_O loaded on ZrO_2_. Small silver oxide particles were revealed as white irregular aggregates on the zirconia surface. The micrographs from SEM–EDX validate the even distribution of silver oxide on the zirconia surface. Results also confirm the data from ICP elemental analysis. The mapping also shows the presence of Ag on ZrO_2_. Furthermore, the morphology of the catalyst from the SEM images also shows the crystallinity and homogeneity of the sample.

### 3.4. BET Surface Area Analysis

**Figure 4:**
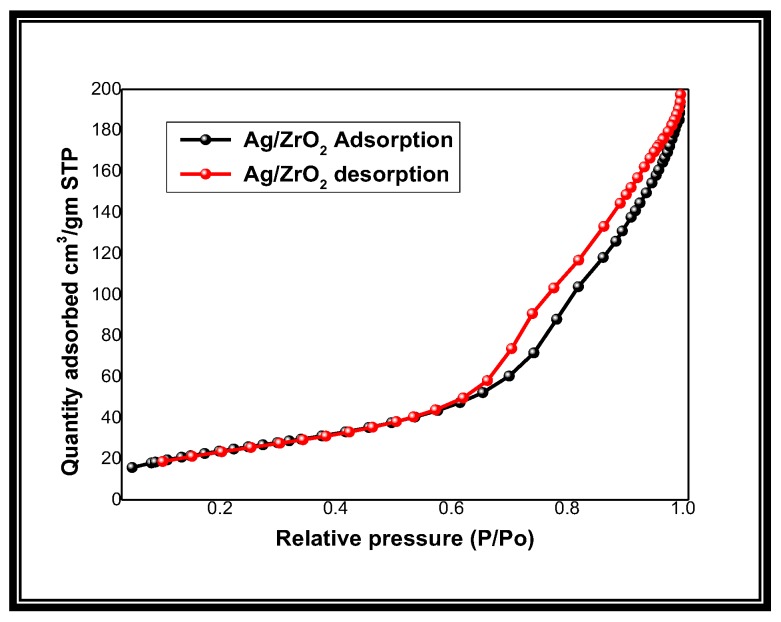
N_2_ adsorption–desorption isotherms of 2.5% Ag_2_O–ZrO_2_ catalyst.

The nitrogen adsorption/desorption isotherm and resulting pore size dispersion of the 2.5% Ag_2_O/ZrO_2_ catalyst material is shown in Figure 4. The catalyst material exhibits a type IV isotherm with the presence of a typical H2 hysteresis loop, which designates the mesoporous nature of the material as per the IUPAC classification. The Barrett-Joyner-Halenda (BJH) pore size distribution describes a mesoporous texture for the material, and the isotherm P/Po range was 0.61–0.98. The BET surface area was measured at 89.52 m^2^/g with a pore volume of 0.330 cm³/g and pore size of 10.3 nm. The ICP study indicates the presence of >1.98 wt % of silver in the catalyst material.

### 3.5. Pyridine Adsorbed FT-IR Spectroscopy

**Figure 5:**
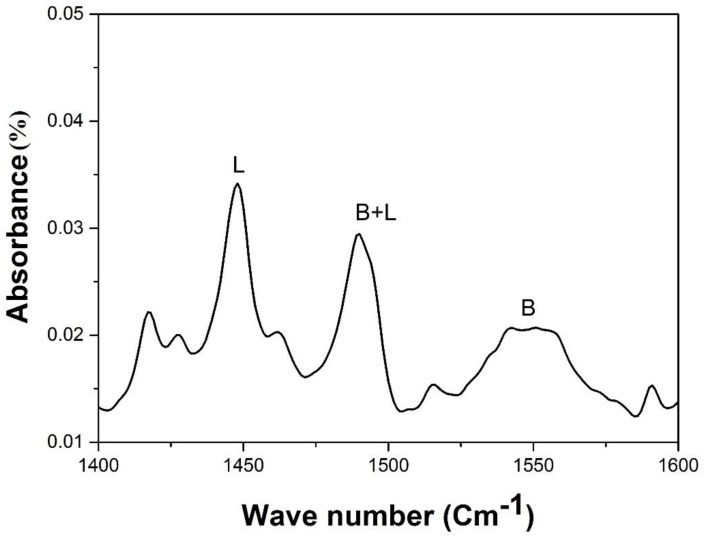
Pyridine FT-IR spectra of 2.5% Ag_2_O/ZrO_2_ catalyst. B = Brønsted acidic sites; L = Lewis acidic sites; B + L = Brønsted and Lewis acidic sites.

The nature of acidic sites on the 2.5% Ag_2_O-loaded ZrO_2_ surface was examined by employing ex situ pyridine FT-IR spectroscopy (Figure 5) [40]. Infrared (IR) spectra were recorded on a Perkin Elmer Precisely equipped with a Universal ATR sampling accessory using a diamond crystal. The powdered material was placed on the crystal and a force of 120 psi was applied to ensure proper contact between the material and the crystal. The spectra were analyzed using Spectrum 100 software. Before recording the IR spectra, pyridine was adsorbed by placing a drop of pyridine on 10 mg of the sample followed by evacuation in air for 1 h at room temperature to remove reversibly adsorbed pyridine on the surface of the catalyst. The IR band at 1540 cm^−1^ confirmed the presence of Brønsted acidic sites (B). The peak observed at 1485 cm^−1^ is attributed to both Brønsted and Lewis acidic sites (B + L). The prominent absorption band at 1450 cm^−1^ is due to the pyridine adsorbed on Lewis acidic sites (L) of the catalyst. The presence of more Lewis acidic sites on the catalyst surface than Brønsted acidic sites is shown in Figure 5. Generally, more Lewis acidic sites are anticipated in the Ag_2_O–ZrO_2_ catalyst due to the availability of vacant metal orbitals on the surface of the catalyst, which are capable of accepting electron pairs from the electron-rich species [41]. Except for the assigned peaks, the other IR bands are mostly less intensive, mainly due to the signal-to-noise ratio, which was unavoidable.

## 4. Reaction Optimization

For optimization of the reaction conditions for a one-pot, three-component reaction involving 2-methoxy benzaldehyde (1 mmol), indane-1,3-dione (1 mmol), and guanidinium hydrochloride (1 mmol), various reaction conditions such as effect of temperature, solvents, and catalysts were investigated. In the absence of solvent and catalyst, no product occurred at RT or under reflux conditions, even after 10 h of reaction (Table 1, entries 1 and 2). The reaction was carried out in ethanol in the presence of various basic catalysts like TEA, pyridine, NaOH, and K_2_CO_3_ at RT; only trace amounts of material were obtained (Table 1, entries 3–6). Reactions with ionic liquids such as (Bmim) BF_4_ or L-proline (Table 1, entries 7 and 8) as a catalyst gave low yields. When the reaction was conducted using acidic catalysts such as AcOH, FeCl_3_, and PTSA, moderate yields of product were attained after 4 h (Table 1, entries 9–11). Consequently, reaction was attempted in presence of pure metal oxide catalysts, such as SiO_2_, ZrO_2_, and Al_2_O_3_, and the reaction showed good yields after 2.0–3.0 h reaction time (Table 1, entries 12–14). Based on the promising outcome with zirconia oxide, to enhance the reaction performance, the efficiencies of different metal oxides loaded on zirconia, such as 2.5% CuO/ZrO_2_, MnO_2_/ZrO_2_, and Ag_2_O/ZrO_2_, were examined. These mixed-oxide heterogeneous catalysts gave very good to excellent yields (82–96%) (Table 1, entries 15–17), while the best result was obtained with Ag_2_O/ZrO_2_ (96% yield, 30 min). Bimetallic metal oxides showed higher activity than their parent metal oxides, presumably due to a better distribution of the active metal on the support and the synergistic activity between the loaded and support materials, providing optimum distribution and increased number of active sites compared to their oxide homologues.

The effect of solvents on the title reaction was investigated in the presence of varied nonpolar solvents. No reaction occurred in n-hexane or toluene. When the reaction was performed in polar aprotic solvents such as THF, DMF, and MeCN, the yield of product was low. In the polar protic solvent MeOH, the yield was good, but lower than that with EtOH. Hence, EtOH was chosen as the solvent for the remainder of the studies. The optimized results are shown in Table 2 (entries 1–8).

**Scheme 2:**
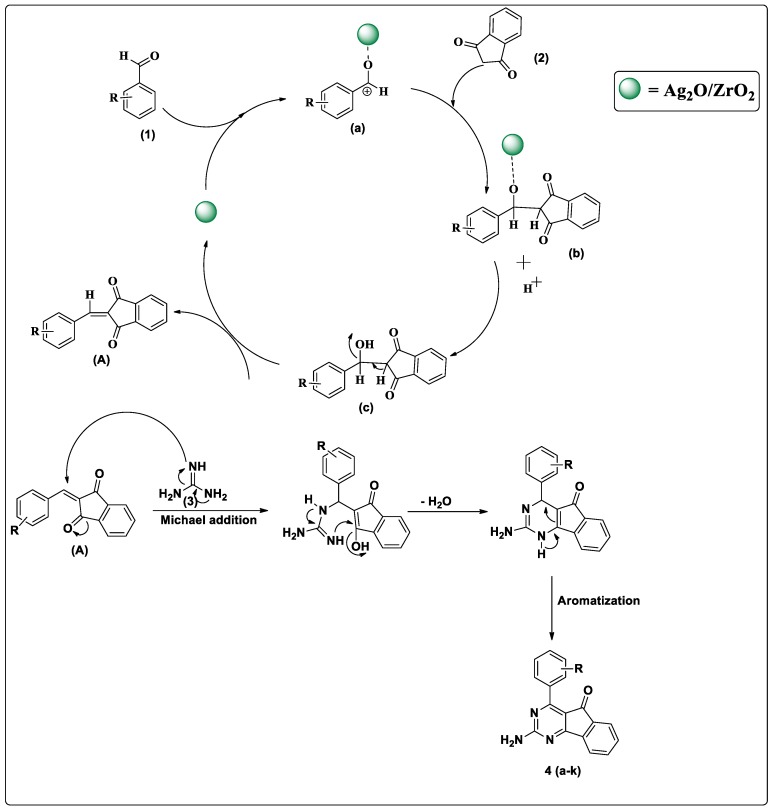
Probable mechanism for the synthesis of novel indenopyrimidine derivatives.

Assuming silver oxide loaded on zirconia as the ideal model catalyst, the contribution of % silver loading on zirconia was investigated at 1.0%, 2.5%, and 5.0% Ag_2_O/ZrO_2_. While 1% Ag loading gave a 90% yield in 45 min, relative to the 2.5% Ag, the 5% Ag neither improved the yield nor decreased the reaction time. The best activity was observed with 2.5% Ag_2_O/ZrO_2_; hence, this was taken as the optimum loading. This could be due to the optimum dispersion of Ag_2_O on ZrO_2_, when compared to 5% Ag_2_O//ZrO_2_, where dispersion was less uniform due to the possible aggregation of silver particles. Hence, catalytic activity was lower compared to the 2.5% loading. The 2.5% loading recorded greater activity than 1% Ag_2_O//ZrO_2_. Possibly, the former had more active sites than the latter (Figure 2). A discussion on the role of the Lewis acidic sites in the reaction is part of the mechanism section (Scheme 2). The efficacy of the reaction, including yield and reaction times for 2.5 wt % Ag_2_O/ZrO_2_, is summarized in Table 3. An increase in catalyst amount from 20 mg to 60 mg improved the yield from 52% to 96% and reduced the reaction time. The increase in the product may be attributed to the comparative increase in the number of available active sites, possibly accelerating the reaction. An increase in the amount of catalyst from 60 mg to 120 mg registered no significant change in the yield of product or reaction time. Hence, 60 mg of the catalyst was considered the ideal amount for the chosen synthesis.

Encouraged by the results, we further explored the applicability of the protocol for other substituted aldehydes under the optimized reaction conditions, by using 10 other substituted aldehydes. The corresponding indenopyrimidines afforded excellent yields in similar reaction times (30 min) (Table 4, entries 1–11). All the reactions, irrespective of electron-withdrawing or electron-donating groups at ortho, meta, or para positions, generally gave excellent yields. All the product molecules were fully characterized by employing ^1^H-NMR, ^13^C-NMR, FT-IR, and HRMS spectral analysis.

A proposed mechanism for the one-pot three-component reaction is outlined in Scheme 2. The presence of Lewis acidic sites on the catalyst surface facilitates the reactants to undergo reaction in a shorter time. It is assumed that in the first step, the Lewis acidic sites with the carbonyl oxygen generate the carbonium ion (**a**) [42]. In a fast reaction with the carbonium ion, the active methylene group affords intermediate (**b**), which desorbs from the catalyst surface by abstracting a proton from the protic solvent, EtOH (**b**), to form (**c**). On further dehydration (**c**), it produces the condensation product (**A**). Next, a Michael addition occurs between the intermediate (**A**) and the guanidinium, followed by cyclization and aromatization, and the transient intermediate yields the target compound—the substituted pyrimidine-5-one derivative. The catalytic efficiency of the Ag_2_O/ZrO_2_ on the title reaction in comparison with other reported catalysts is summarized in the Table 5.

## 5. Reusability of Catalyst

**Figure 6:**
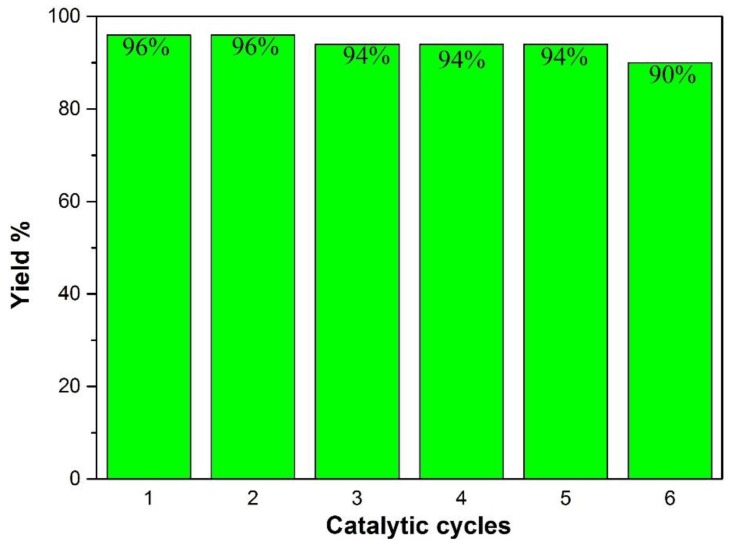
Recyclability of the Ag_2_O/ZrO_2_ catalyst.

The main objective and attraction of heterogeneous catalysts are its reusability. We thus examined the recovery and reusability of the Ag_2_O/ZrO_2_ catalyst. The solid catalyst from the reaction mixture was separated by simple filtration under vacuum, followed by washing with acetone solvent and drying at 100 °C for 3 h. The recovered catalyst was reused in subsequent reactions. Six runs in successive reactions gave yields without significant loss in product yield (Figure 6).

## 6. Conclusions

In conclusion, we report a simple and green protocol for the synthesis of indenopyrimidines by a three-component reaction. All the reactions involving the reaction of 11 different aromatic aldehydes with 1,3-indandione and guanidinium hydrochloride using (2.5%) silver loaded on a zirconia catalyst gave excellent yields (90–96%). The proposed catalyst proved efficient, stable, and reusable. This method offers easy workup, excellent selectivity, and high yields in short reaction times at room temperature using ethanol, a green solvent. All the products were purified by recrystallization from ethanol. This method needs no chromatographic separation. Consequently, the use of volatile and hazardous solvents has been evaded. This method is useful for synthesizing various privileged pyrimidine scaffolds in short times in a one-pot strategy under green conditions.

## Figures and Tables

**Table 1:** Effect of catalysts on the synthesis of **4a** ^a^.

Entry	Catalyst	Solvent	Condition	Time (h)	Yield (%) ^b^
1	--	--	RT	10	--
2	--	--	Reflux	10	--
3	TEA	EtOH	RT	8.0	9
4	Pyridine	EtOH	RT	8.5	13
5	NaOH	EtOH	RT	7.5	25
6	K_2_CO_3_	EtOH	RT	7.0	19
7	(Bmim)BF_4_	EtOH	RT	10	23
8	L-proline	EtOH	RT	10	27
9	AcOH	EtOH	RT	5.0	43
10	FeCl_3_	EtOH	RT	4.5	50
11	PTSA	EtOH	RT	5.0	45
12	SiO_2_	EtOH	RT	2.5	62
13	ZrO_2_	EtOH	RT	2.0	79
14	Al_2_O_3_	EtOH	RT	3.0	59
15	2.5% CuO/ZrO_2_	EtOH	RT	1.5	82
16	2.5% MnO_2_/ZrO_2_	EtOH	RT	1.0	87
17	2.5% Ag_2_O/ZrO_2_	EtOH	RT	0.20	96

^a^ All products were characterized by ^1^H-NMR, ^13^C-NMR, HRMS, and FT-IR spectral analysis. ^b^ Isolated yields after recrystallization. -- No reaction.

**Table 2:** Optimization of various solvent conditions for the model reaction ^a^.

Entry	Solvent	Time (min)	Yield (%)
1	No solvent	120	--
2	n-hexane	120	--
3	toluene	90	--
4	THF	75	10
5	DMF	65	18
6	MeCN	60	25
7	MeOH	45	81
8	EtOH	30	96

^a^ Reaction conditions: arylaldehyde (**1**), (1 mmol), 1,3-Indandione (1 mmol) (**2**), and guanidinium hydrochloride (**3**) (1 mmol) and solvent (5 mL) were stirred at room temperature. -- No isolated yields.

**Table 3:** Optimization of various weight % for the model reaction with 2.5% Ag_2_O/ZrO_2_ catalyst ^a^.

Entry	Catalyst (mg)	Time (min)	Yield (%)
1	20	100	56
2	40	50	79
3	60	30	96
4	80	30	96
5	100	30	96
6	120	40	96

^a^ Reaction conditions: arylaldehyde (**1**) (1 mmol), 1,3-indanedione (**2**) (1 mmol), and guanidine hydrochloride (**3**) (1 mmol) and catalyst and solvent (5 mL) were stirred at room temperature.

**Table 4:** Synthesis of novel functionalized pyridine derivatives by 2.5% Ag_2_O/ZrO_2_ catalyst ^a^.

Entry	R	Product	Yield * (%)	m.p. (°C)	Lit. m.p. (°C)
1	2-OMe	**4a**	94	119–121	-
2	4-OMe	**4b**	93	136–137	100 [20]
3	2,3-(OMe)_2_	**4c**	94	183–184	-
4	2,5-(OMe)_2_	**4d**	92	200–201	-
5	2-Br	**4e**	94	197–198	-
6	2-F	**4f**	92	239–241	-
7	3,4-(OMe)_2_	**4g**	96	176–178	178 [20]
8	3-OH	**4h**	95	246–248	-
9	4-Br	**4i**	94	221–223	210 [20]
10	4-Cl	**4j**	90	239–241	244 [20]
11	4-Et	**4k**	94	204–206	-

^a^ Reaction conditions: arylaldehyde (**1**) (1 mmol), 1,3-indanedione (**2**) (1 mmol), and guanidine hydrochloride (**3**) (1 mmol), catalyst (60 mg), and ethanol solvent (5 mL) were stirred at room temperature. R = substituted benzaldehydes. - New compounds/no literature (lit.) data. * = Isolated yields after recrystallization.

**Table 5:** A comparison table with various other catalysts for synthesizing pyrimidine derivatives.

Catalyst	Solvent	Reaction Condition	Yield (%) [Ref]
NaOH	EtOH	Reflux, 6–10 h	81–94 [20]
NaOH	EtOH	Reflux, 7–8.4 h	75–86 [21]
NaOH	EtOH	Reflux, 0.5–1 h	85–94 [22]
NaOMe	EtOH	Reflux, 10–14 h	60–70 [23]
α-Fe_2_O_3_-MCM-41-P	Solvent free	80 °C, 1 h	82–95 [25]
Uranyl acetate/succinimide sulfonic acid	Solvent free	90 °C, 4 h	75–96 [26]
2.5% Ag_2_O–ZrO_2_	EtOH	RT, 30 min	90–96 [Present Work]

## Supplementary Materials

Supplementary Materials are available online.
