# Supplementary material for: Ag2O on ZrO2 as a Recyclable Catalyst for Multicomponent Synthesis of Indenopyrimidine Derivatives
Source: Molecules. 2018 Jul 5;23(7):1648. doi: 10.3390/molecules23071648 (PMC6100188; doi:10.3390/molecules23071648)
Supplement: Supplementary file 1 [file molecules-23-01648-s001.pdf]

molecules

Ag_2O on ZrO_2 as a Recyclable Catalyst for Multicomponent Synthesis of Indenopyrimidine Derivatives

Sandeep V.H.S. Bhaskaruni, Suresh Maddila, Werner E. van Zyl, Sreekantha B. Jonnalagadda*

*School of Chemistry & Physics, University of KwaZulu-Natal, Westville Campus,

Chiltern Hills, Durban-4000, South Africa.

Contents	Pages
Catalyst Instrumentation Details (SI – I)	2
Materials, Methods, and Instruments (SI – I)	2
All Synthesized Spectral Information (SI – II)	3-24

Catalyst Instrumentation Details [Supporting Information (SI–I)]

Employing a Bruker D8 Advance instrument (Cu K radiation source with a wave length of 1.5406 Å), the X-ray diffraction data related to the structural phases of the catalyst were acquired. Using a Jeol JEM-1010 electron microscope and a JEOL JSM-6100 microscope, the TEM and SEM analysis data were recorded. iTEM software was used to analyse the TEM data and images. Employing an X-ray analyser (energy-dispersive), EDX analysis on the SEM images was conducted.

Experimental Section [Supporting Information (SI–I)]

All chemicals and reagents required for the reaction were of analytical grade and were used without any further purification. A Bruker AMX 400 MHz NMR spectrometer was used to record the ^{1}H NMR, and ^{13}C NMR spectral values. Top spin 3.5 software was used to analyse the 1H NMR, and 13C NMR spectral data. Infrared (IR) spectra were recorded on a Perkin Elmer 100 Fourier transform infrared spectrophotometer. High-resolution mass data were obtained using a Bruker micro TOF-Q II ESI instrument operating at ambient temperature. The DMSO−d_6 solution was utilized for this while TMS served as the internal standard. TMS was further used as an internal standard for reporting the all chemical shifts in δ (ppm). The purity of all the reaction products was confirmed by TLC using aluminum plates coated with silica gel (Merck Kieselgel 60 F254).

Synthesized Spectral Information [Supporting Information SI–II]

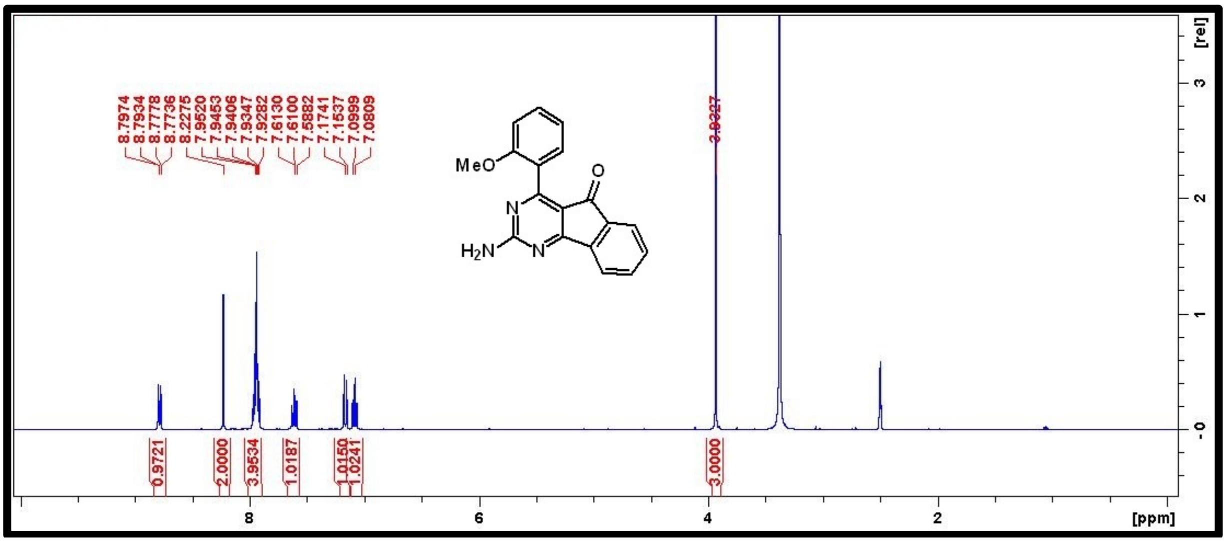

^{1}H NMR spectra of compound **4a**

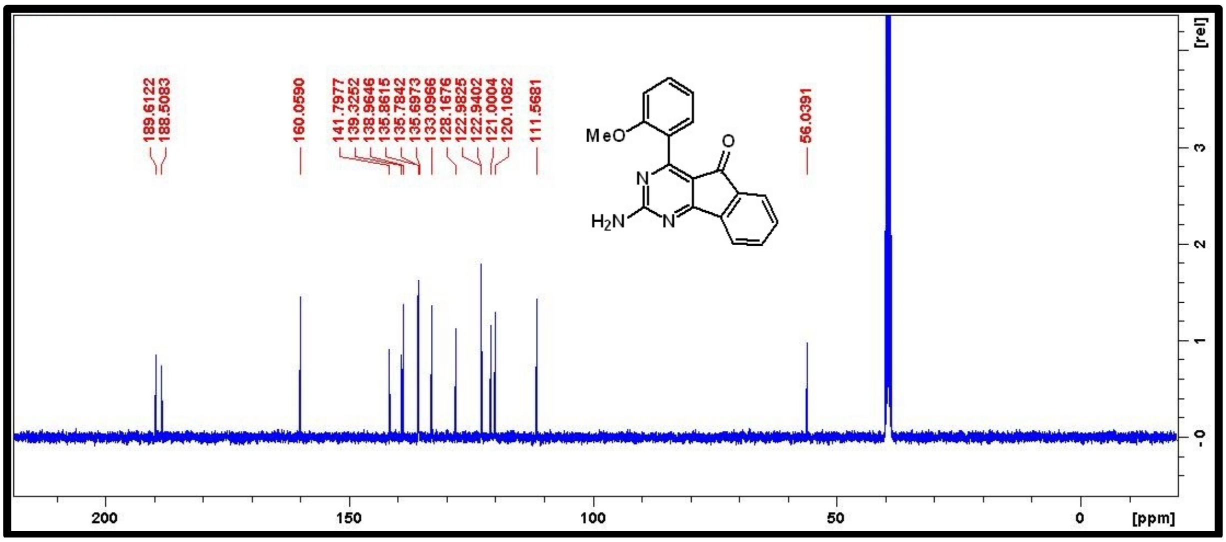

^{13}C NMR spectra of compound **4a**

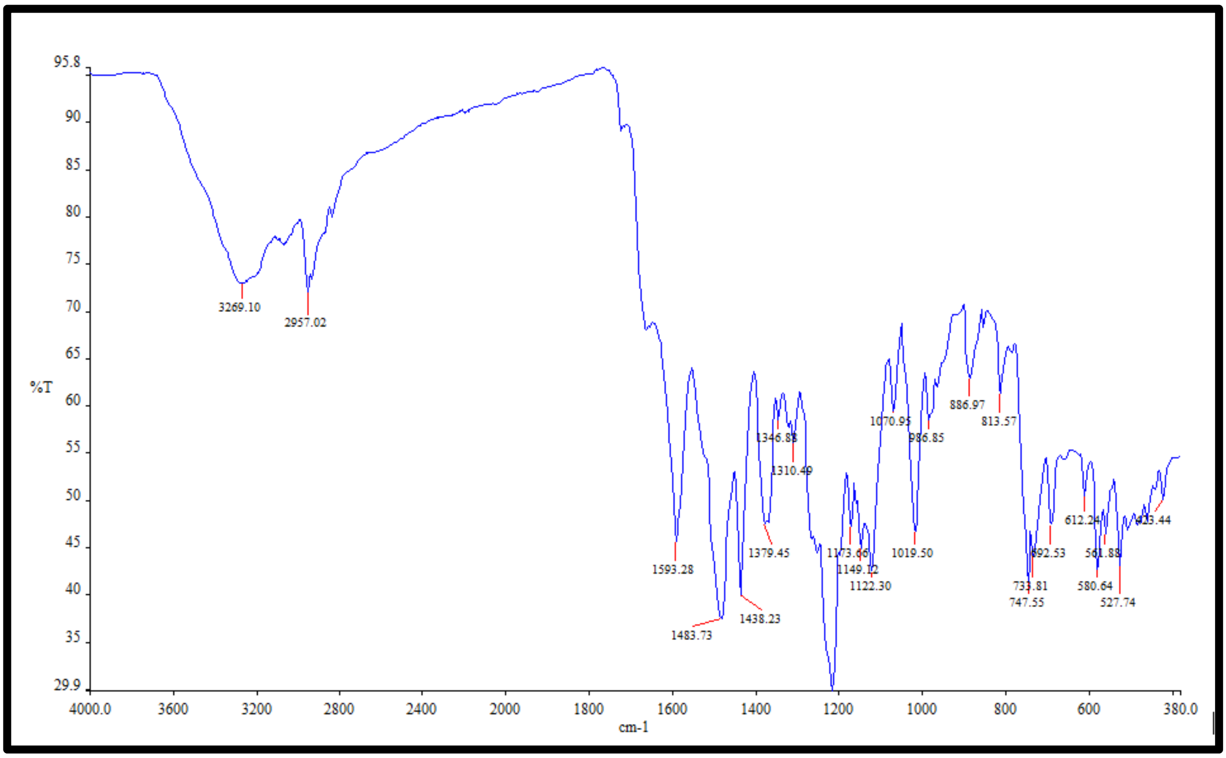

FT-IR spectra of compound **4a**

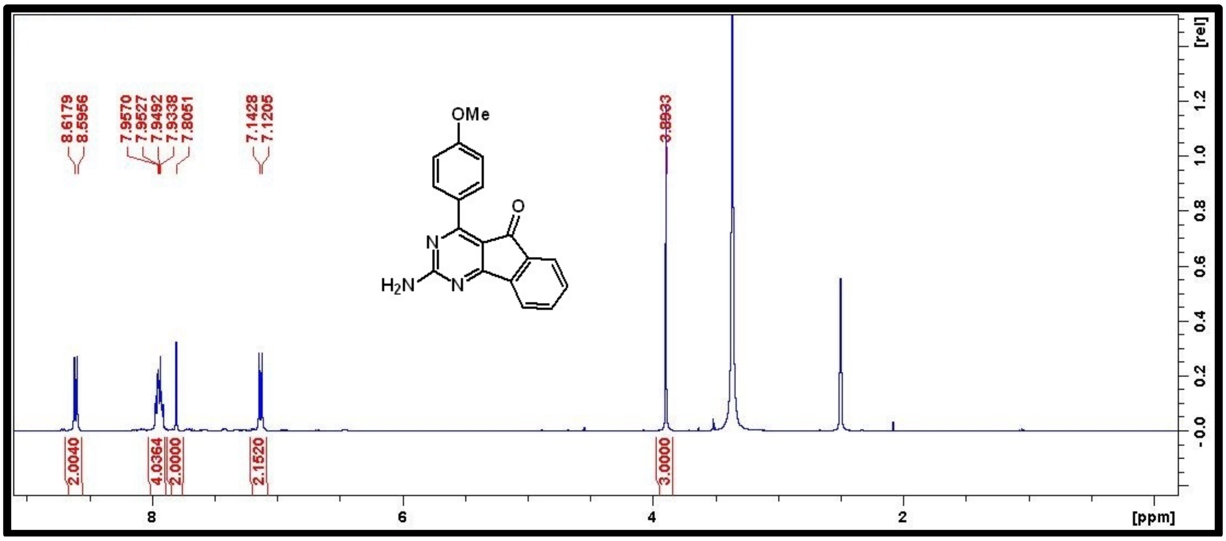

^{1}H NMR spectra of compound **4b**

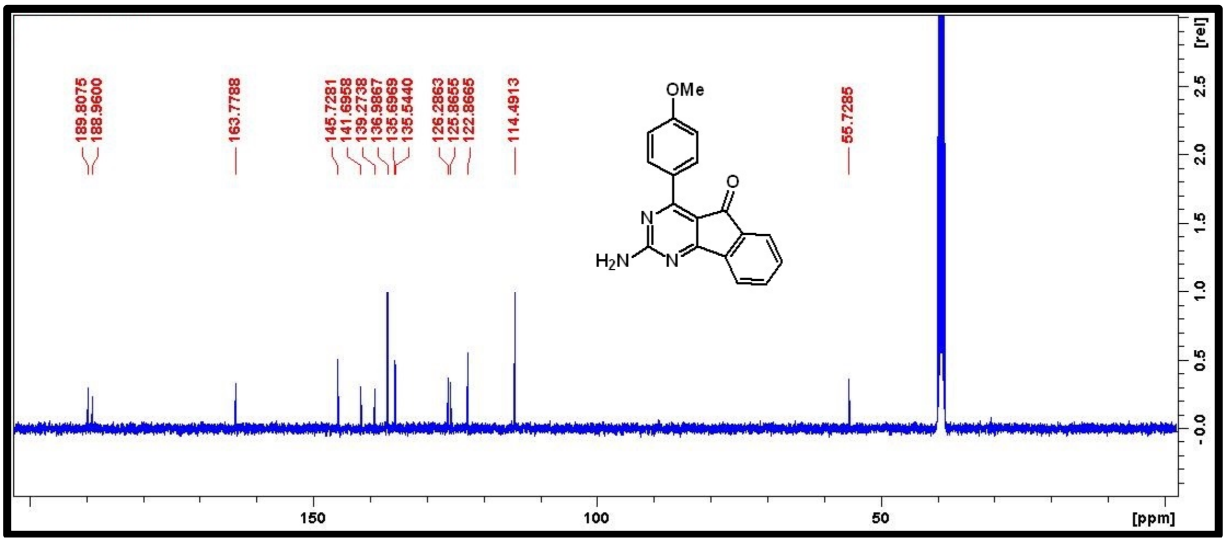

^{13}C NMR spectra of compound **4b**

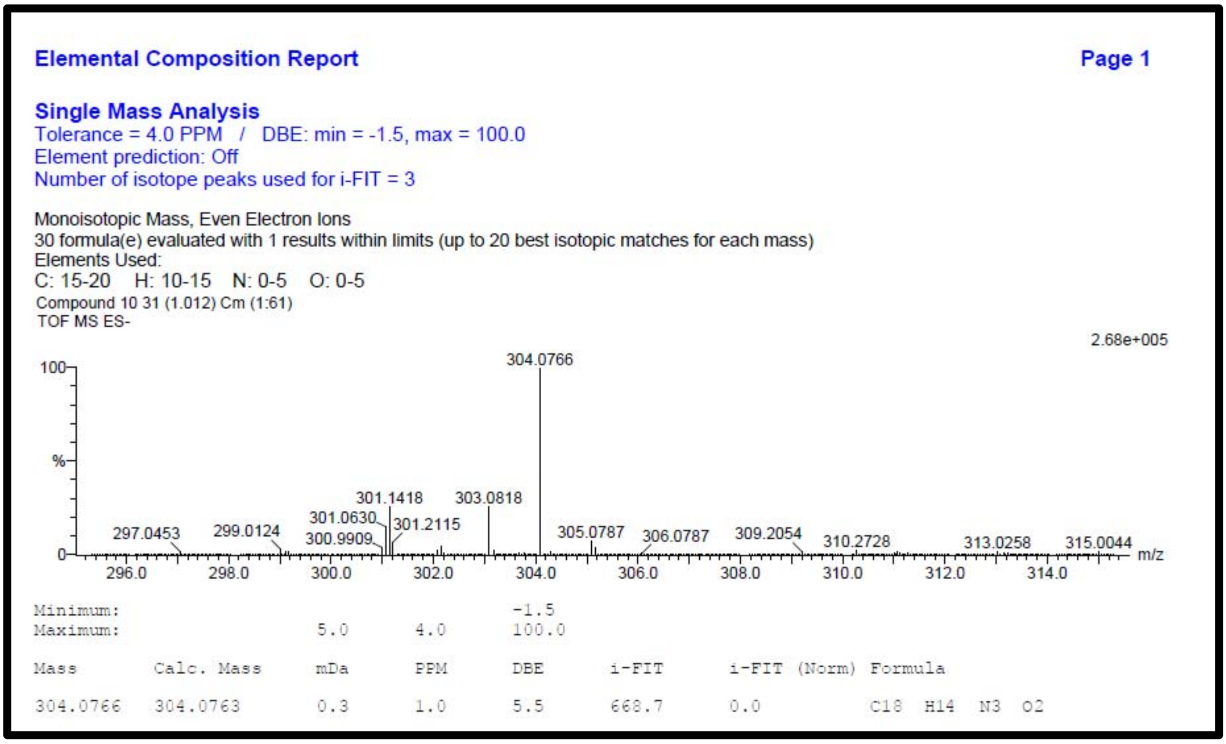

HRMS spectra of compound **4b**

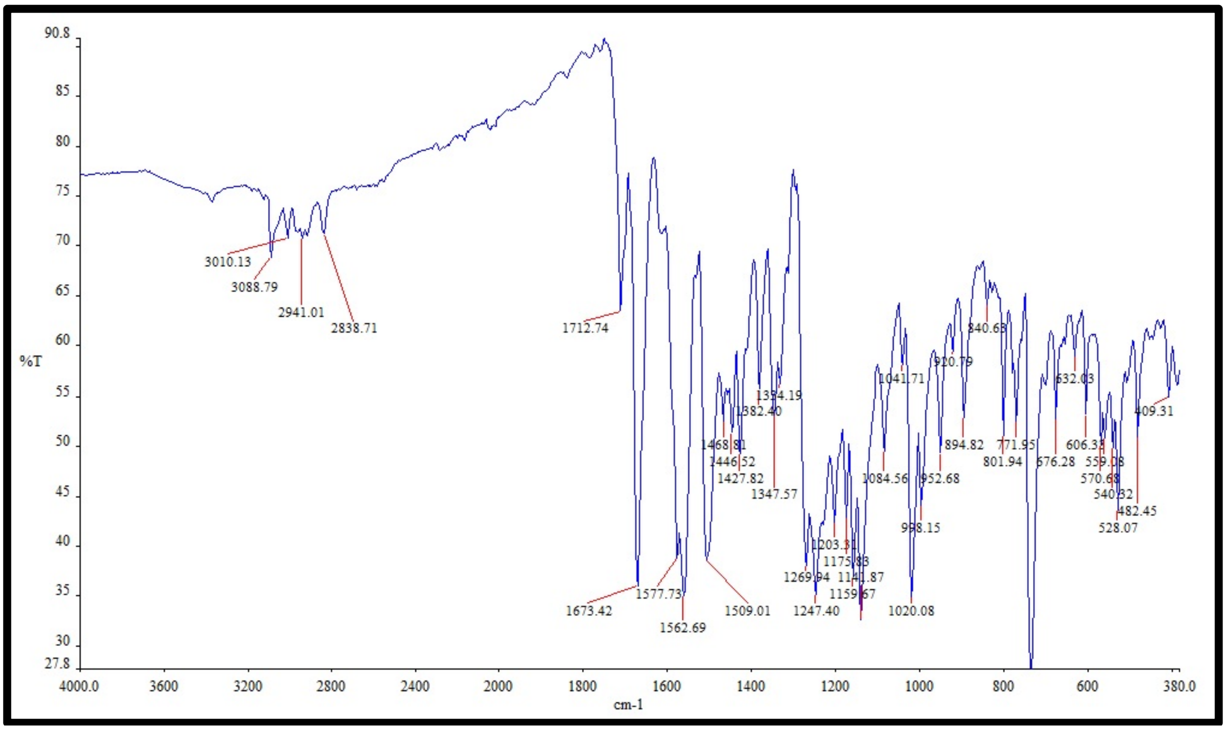

FT-IR spectra of compound **4b**

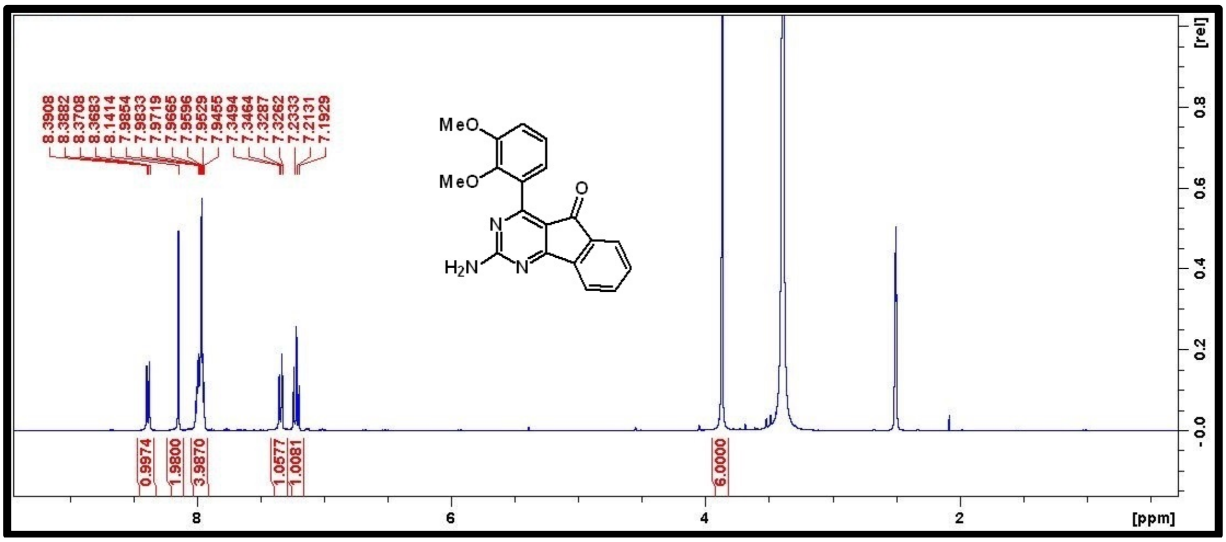

^{1}H NMR spectra of compound **4c**

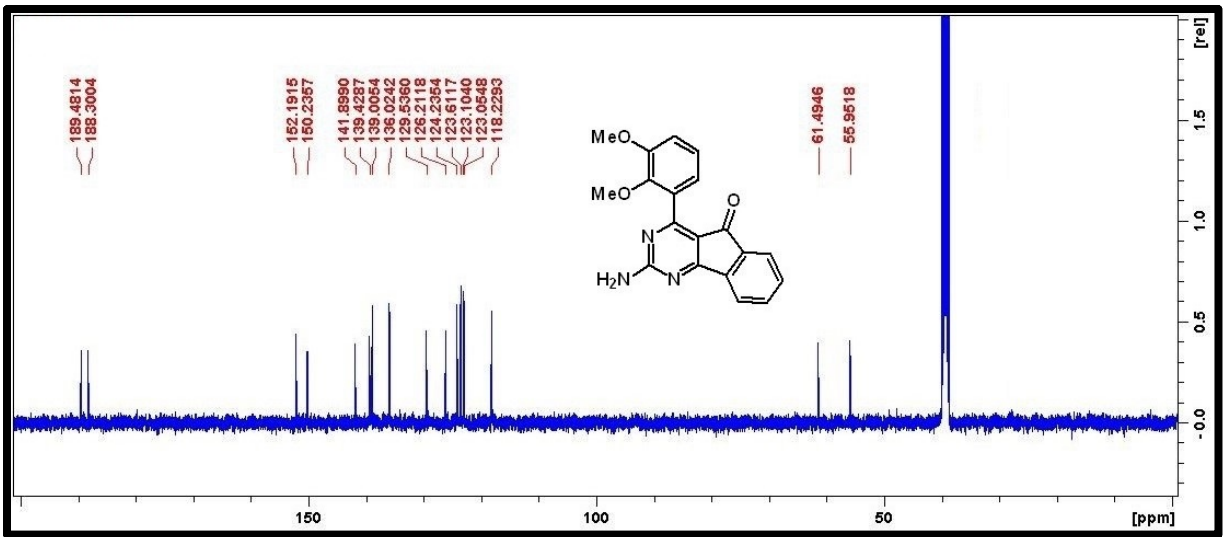

^{13}C NMR spectra of compound **4c**

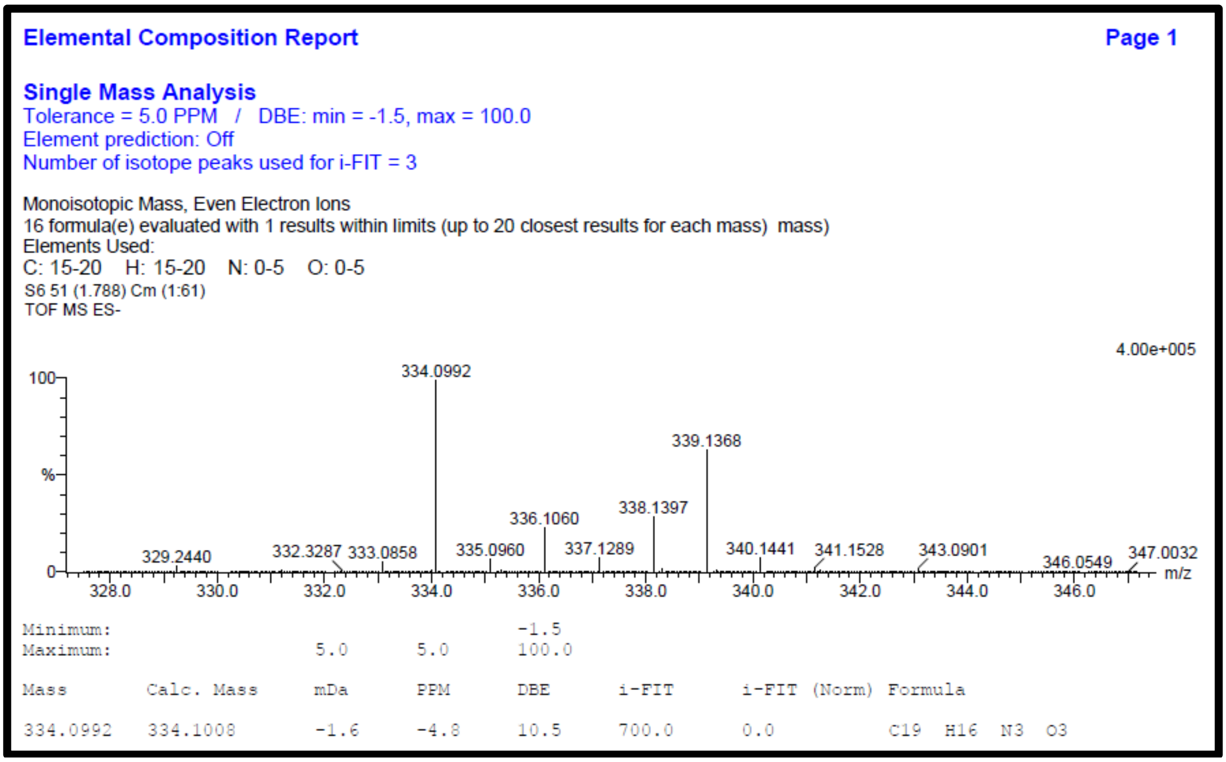

HRMS spectra of compound **4c**

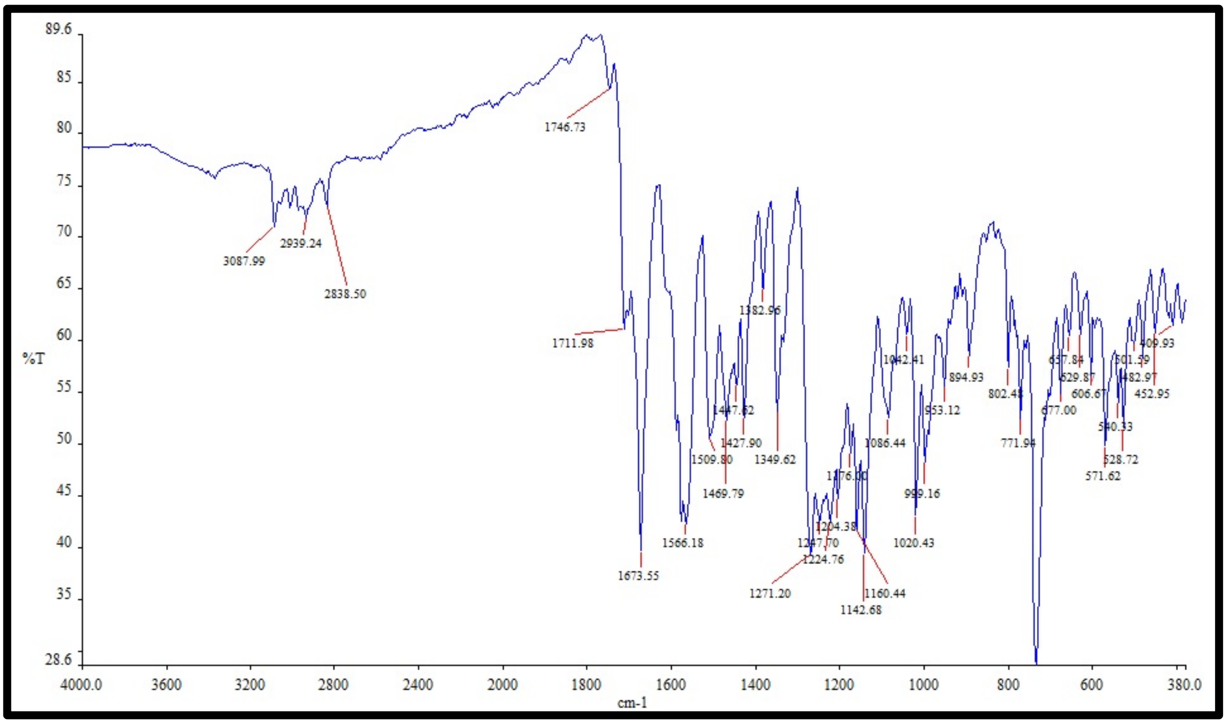

FT-IR spectra of compound **4c**

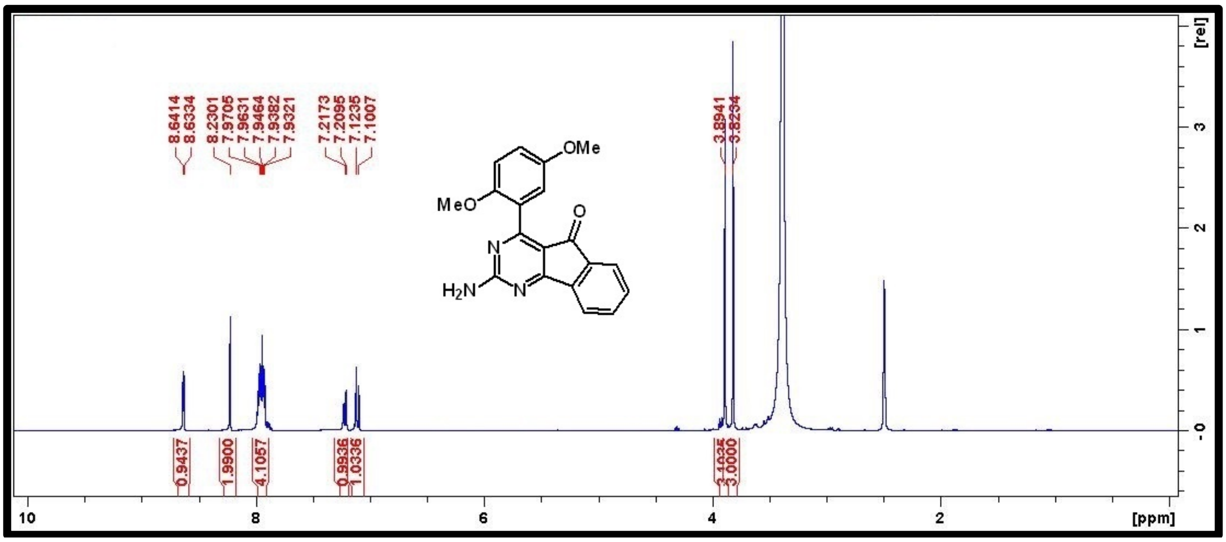

^{1}H NMR spectra of compound **4d**

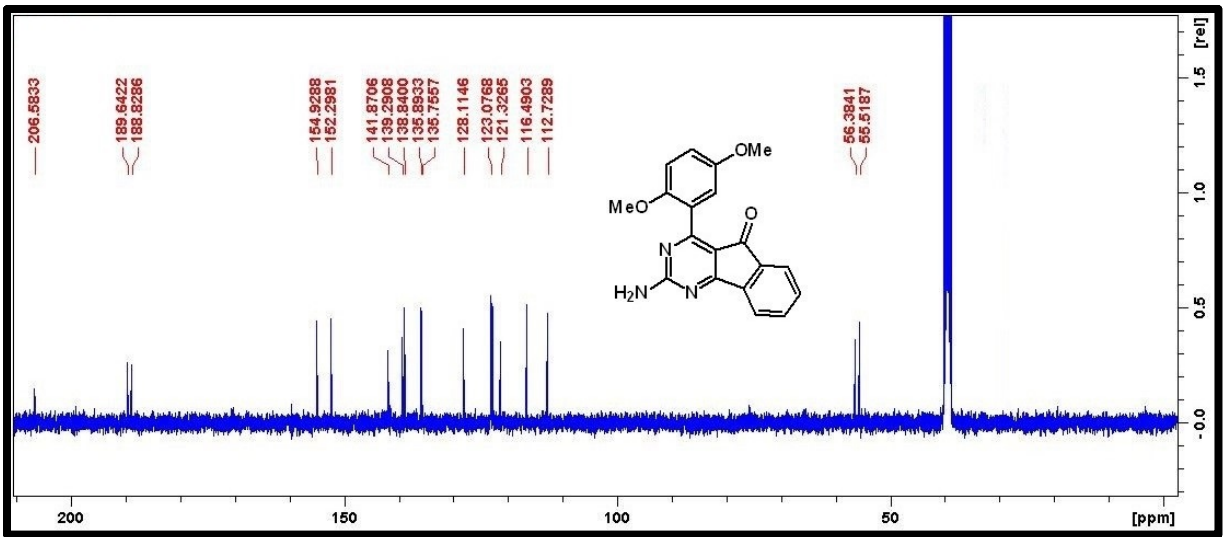

^{13}C NMR spectra of compound **4d**

Elemental Composition Report **Page 1**

Single Mass Analysis
Tolerance = 5.0 PPM / DBE: min = -1.5, max = 100.0
Element prediction: Off
Number of isotope peaks used for i-FIT = 3

Monoisotopic Mass, Even Electron Ions
16 formula(e) evaluated with 1 results within limits (up to 20 closest results for each mass)
Elements Used:
C: 15-20 H: 15-20 N: 0-5 O: 0-5
S3 54 (1.788) Cm (1:61)
TOF MS ES-

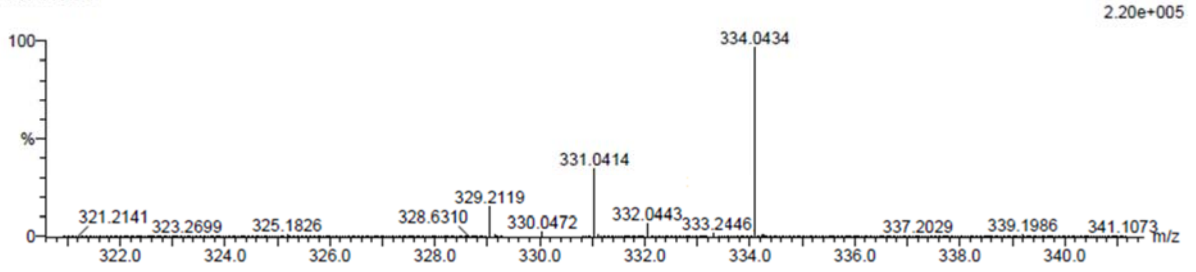

Minimum:				-1.5			
Maximum:		5.0	5.0	100.0			
Mass	Calc. Mass	mDa	PPM	DBE	i-FIT	i-FIT (Norm)	Formula
334.0434	334.0441	-0.7	-2.1	12.5	652.1	0.0	C19 H16 N3 O3

HRMS spectra of compound **4d**

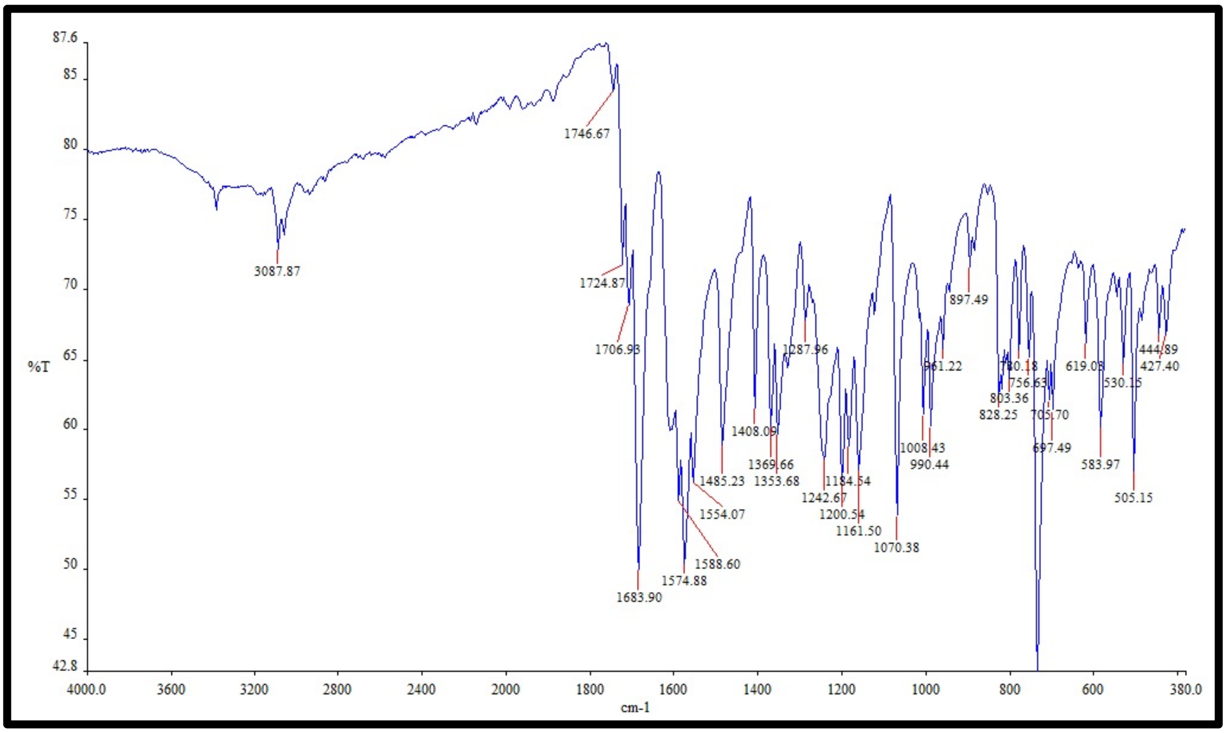

FT-IR spectra of compound **4d**

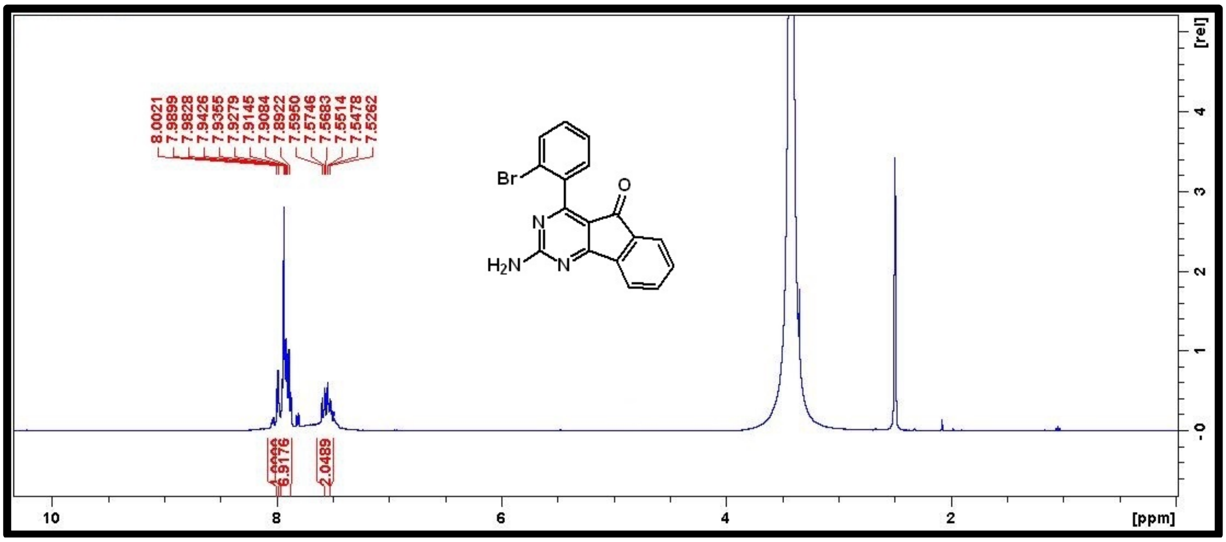

^{1}H NMR spectra of compound **4e**

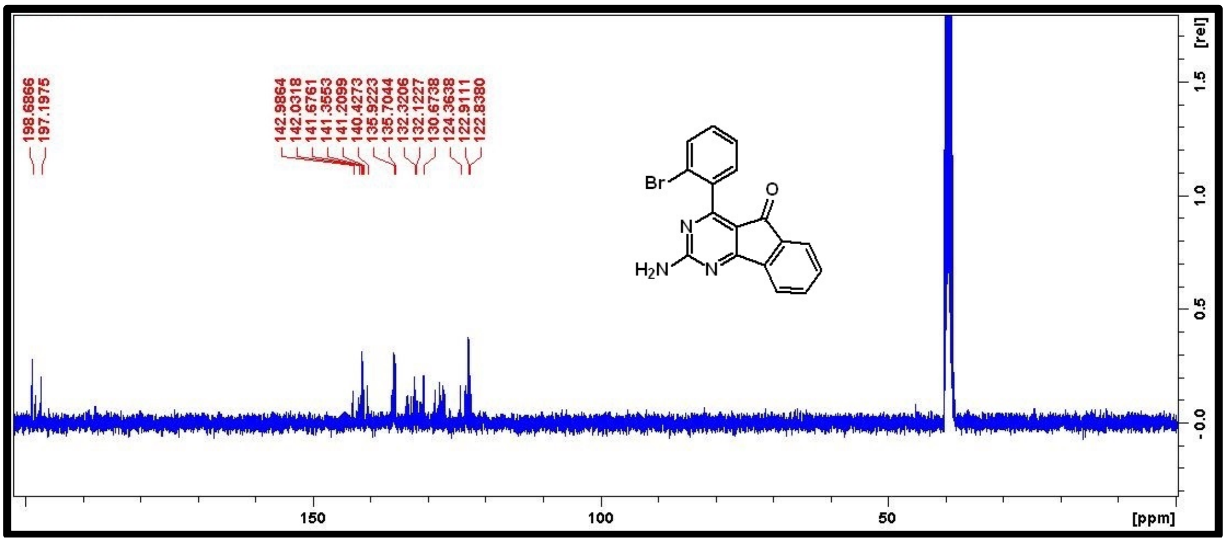

^{1}C NMR spectra of compound **4e**

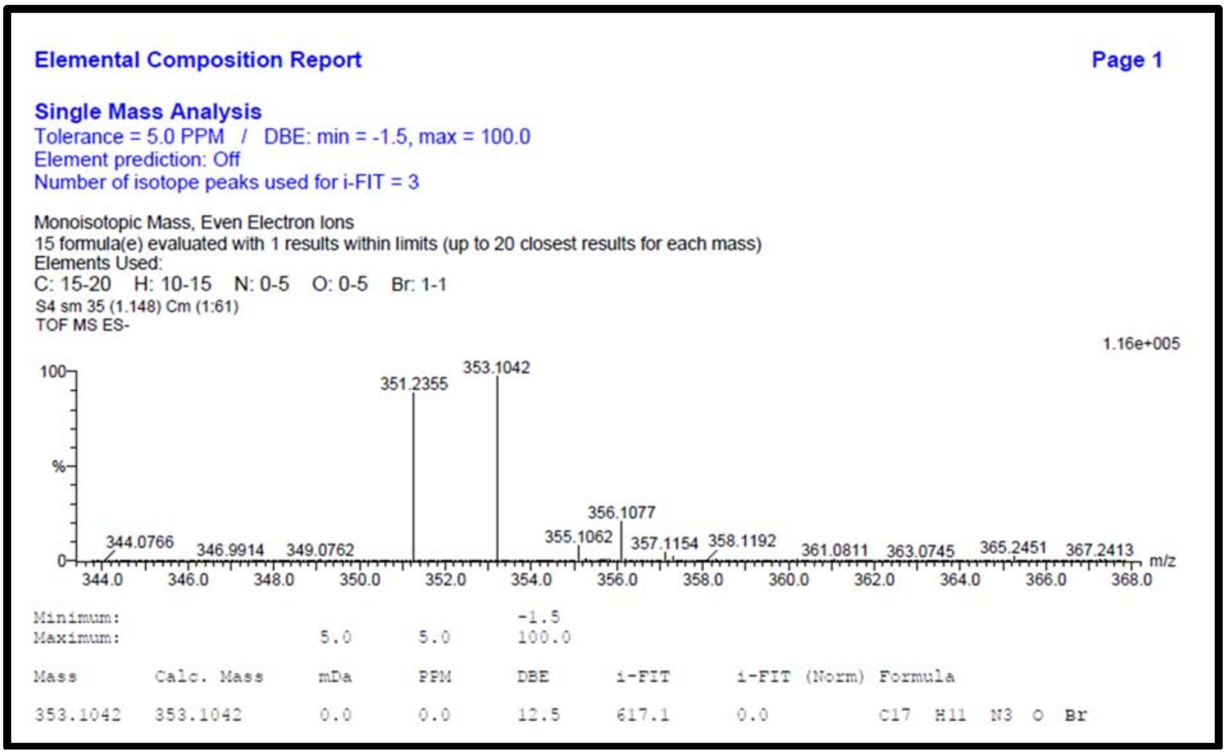

HRMS spectra of compound **4e**

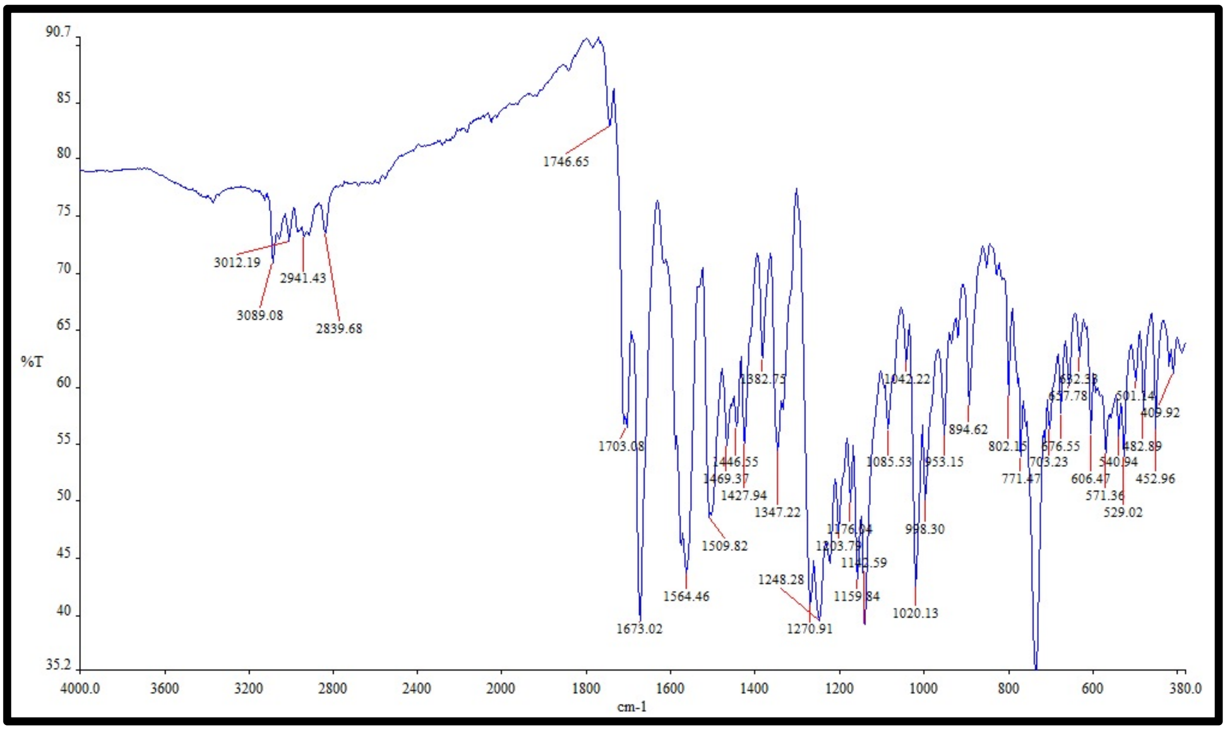

FT-IR spectra of compound **4e**

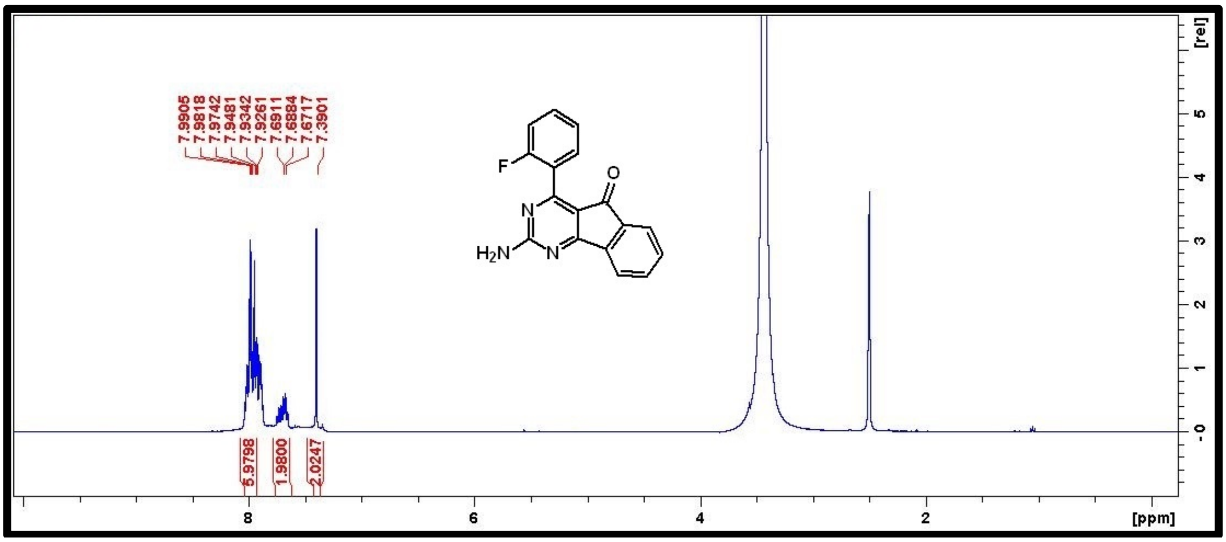

^{1}H NMR spectra of compound **4f**

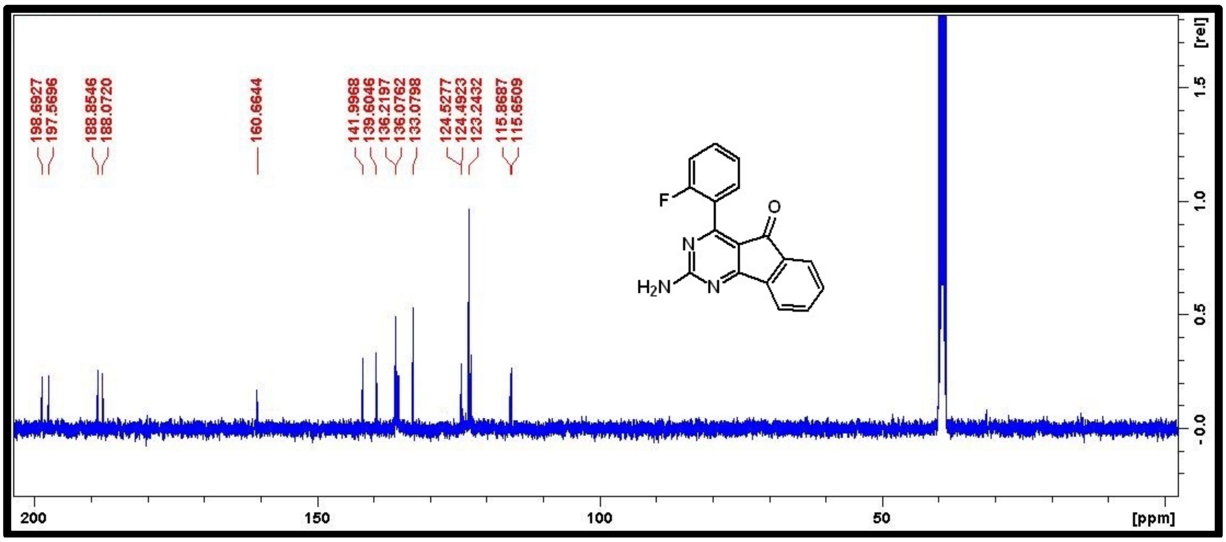

^{13}C NMR spectra of compound **4f**

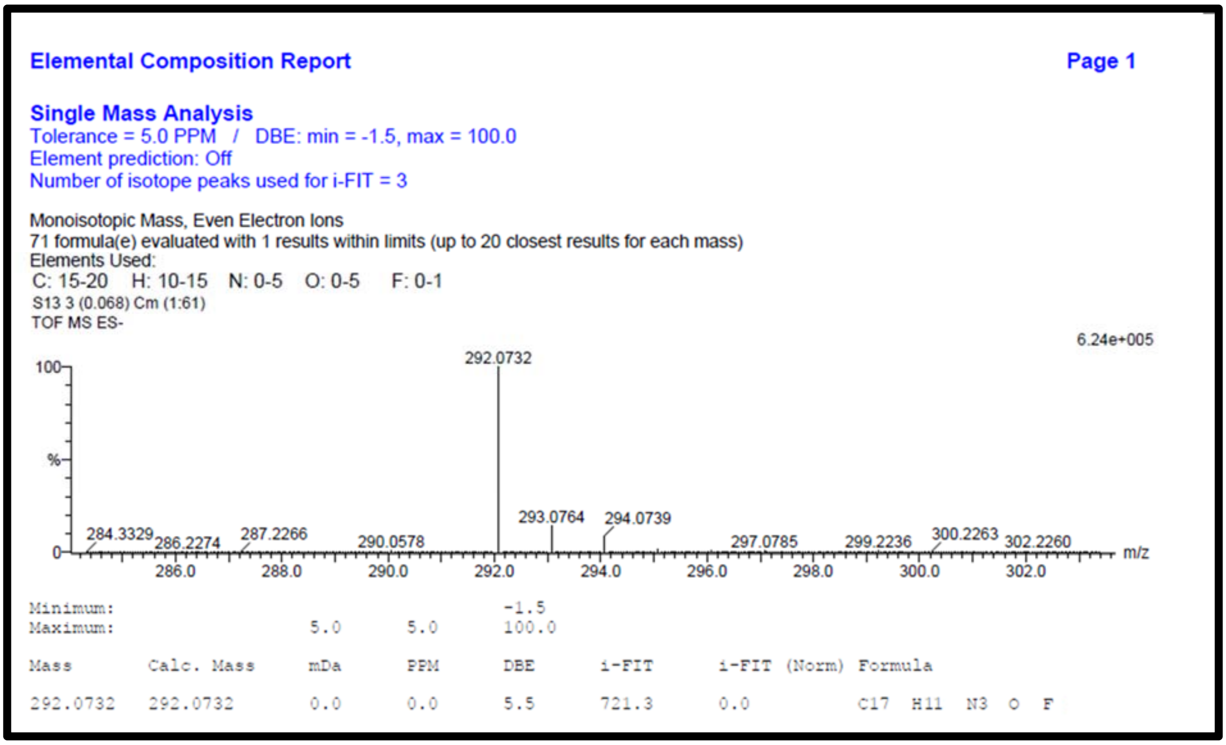

HRMS spectra of compound **4f**

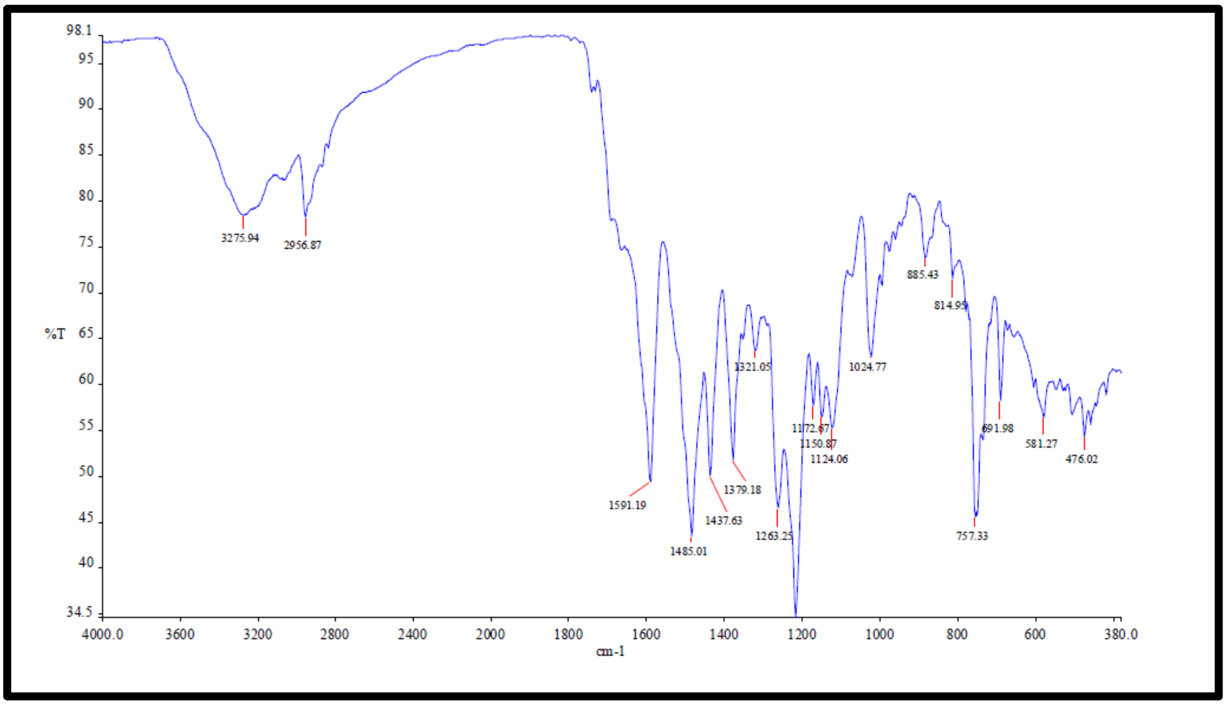

FT-IR spectra of compound **4f**

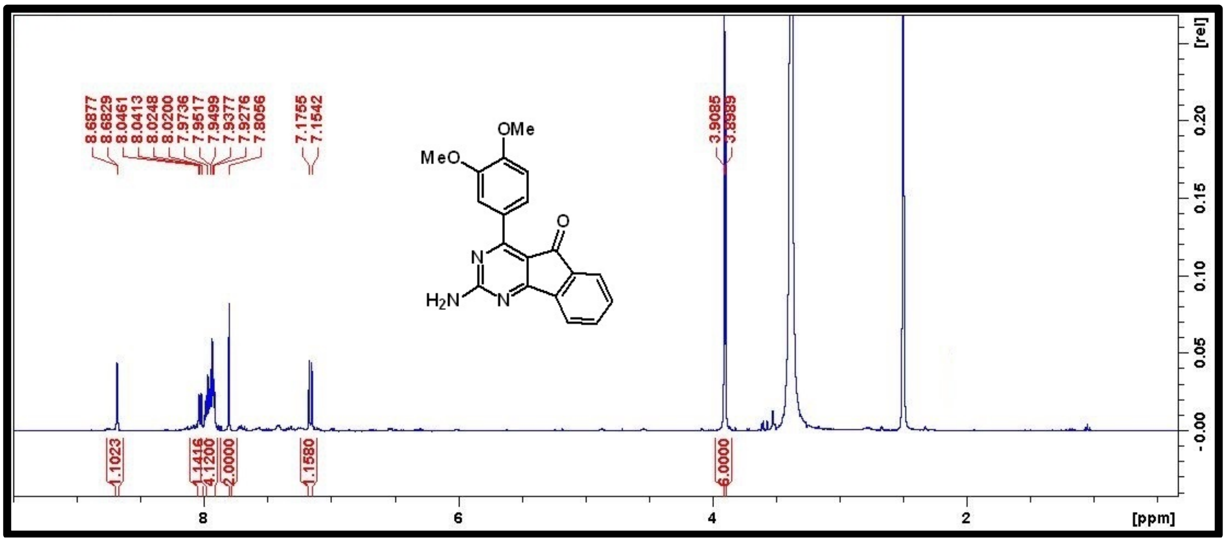

FT-IR spectra of compound **4g**

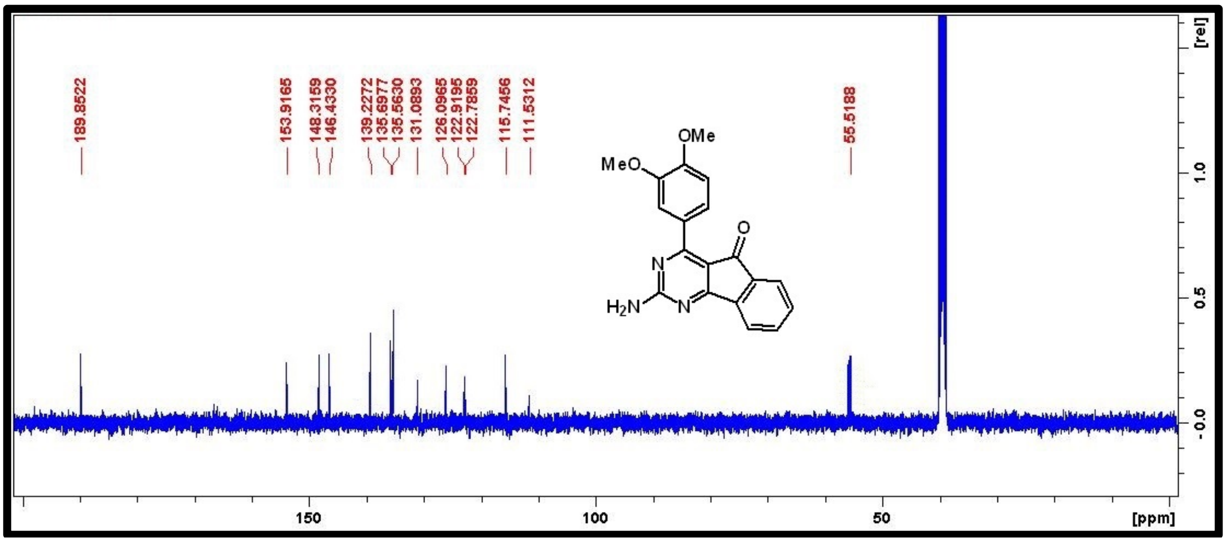

^{13}C NMR spectra of compound 4g

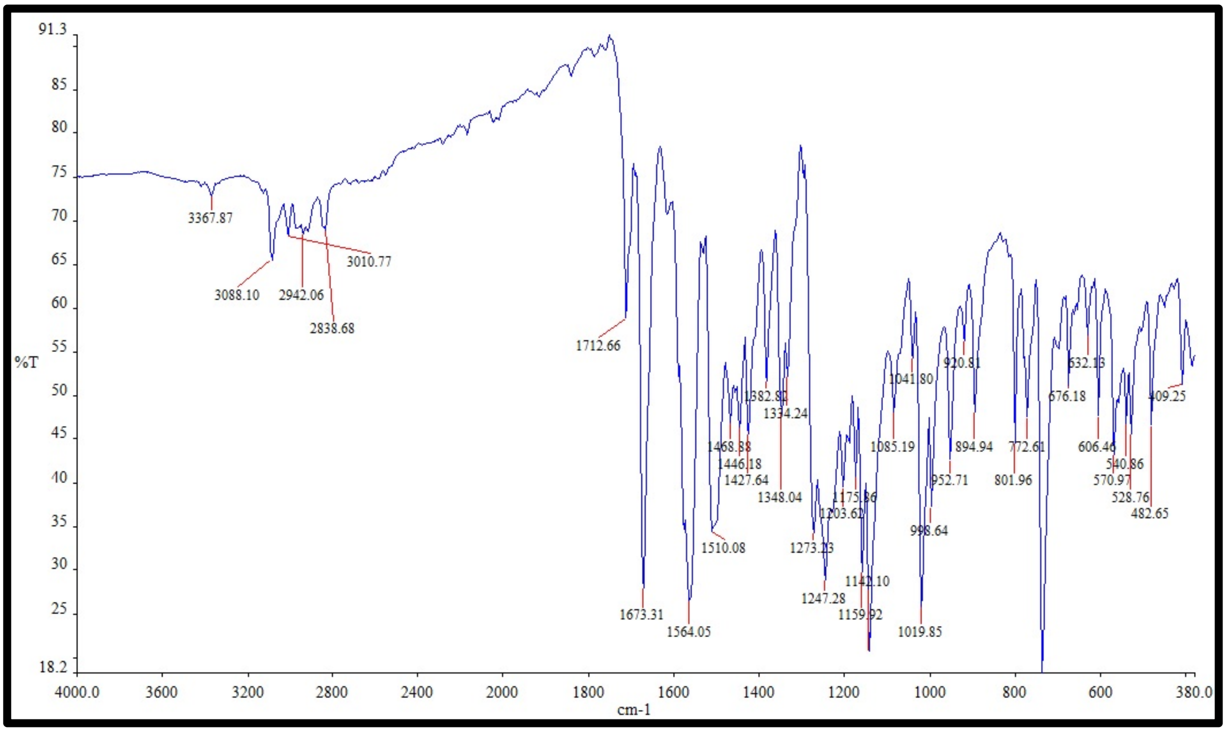

FT-IR spectra of compound **4g**

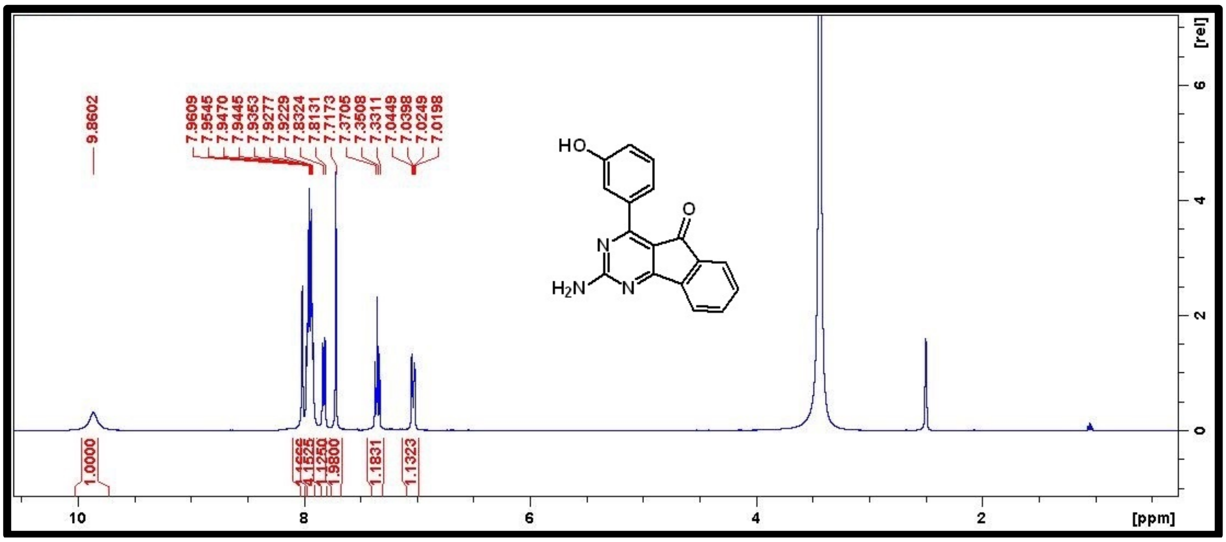

^{1}H NMR spectra of compound **4h**

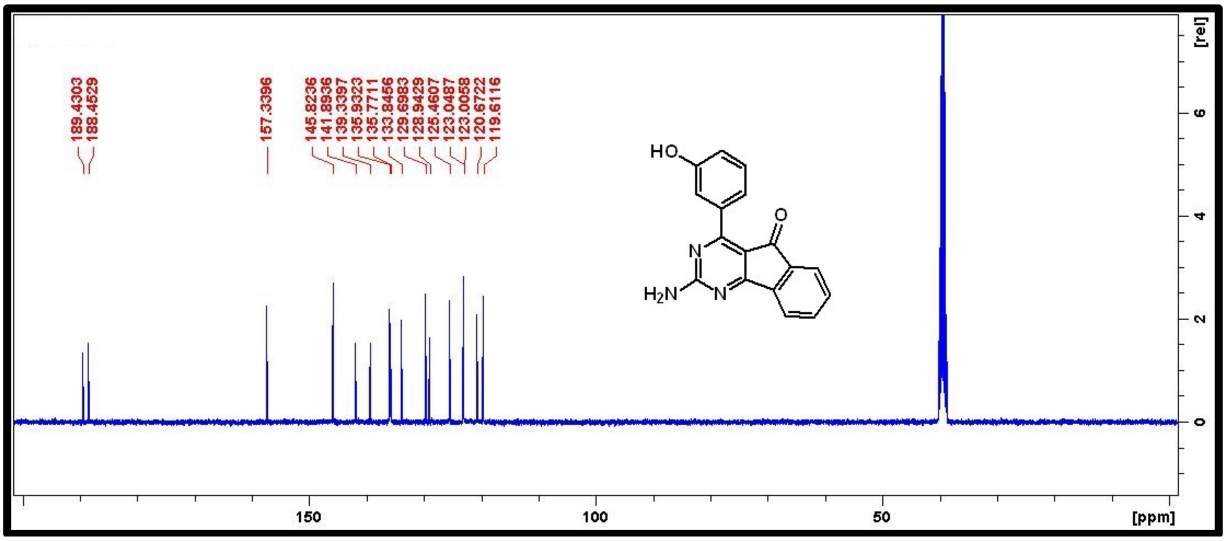

^{13}C NMR spectra of compound **4h**

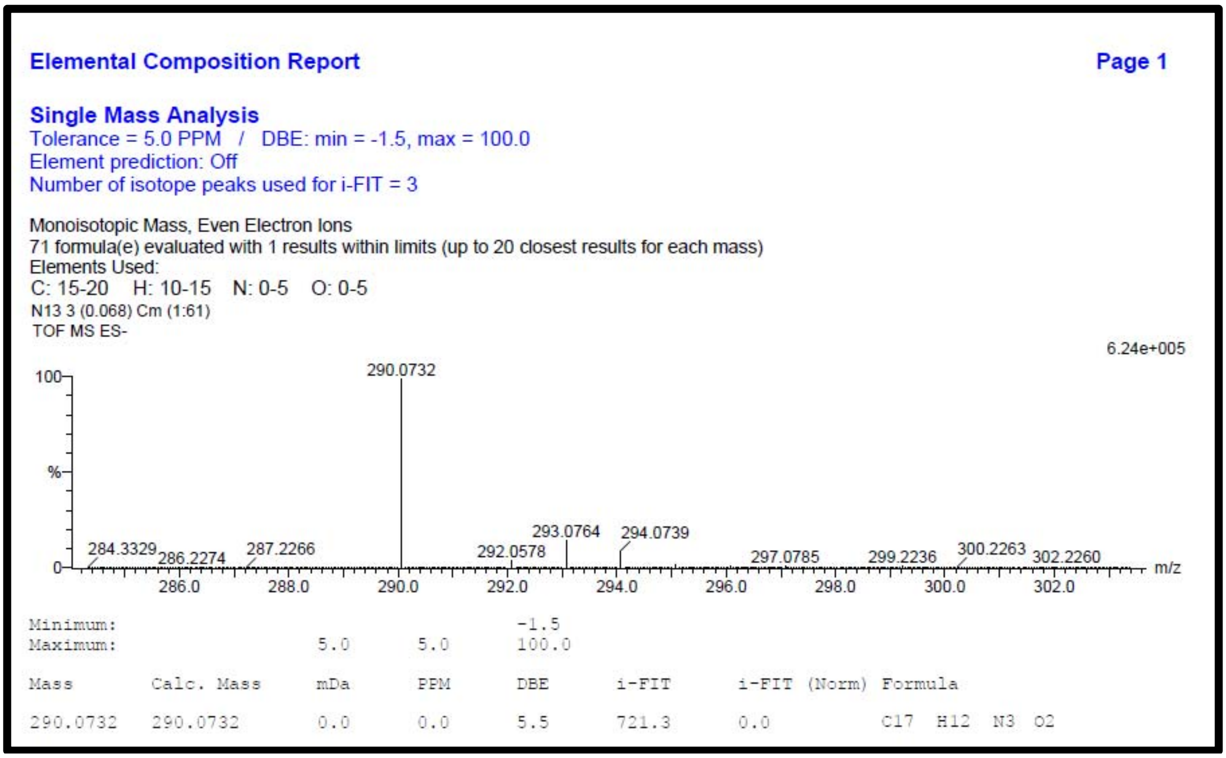

HRMS spectra of compound **4h**

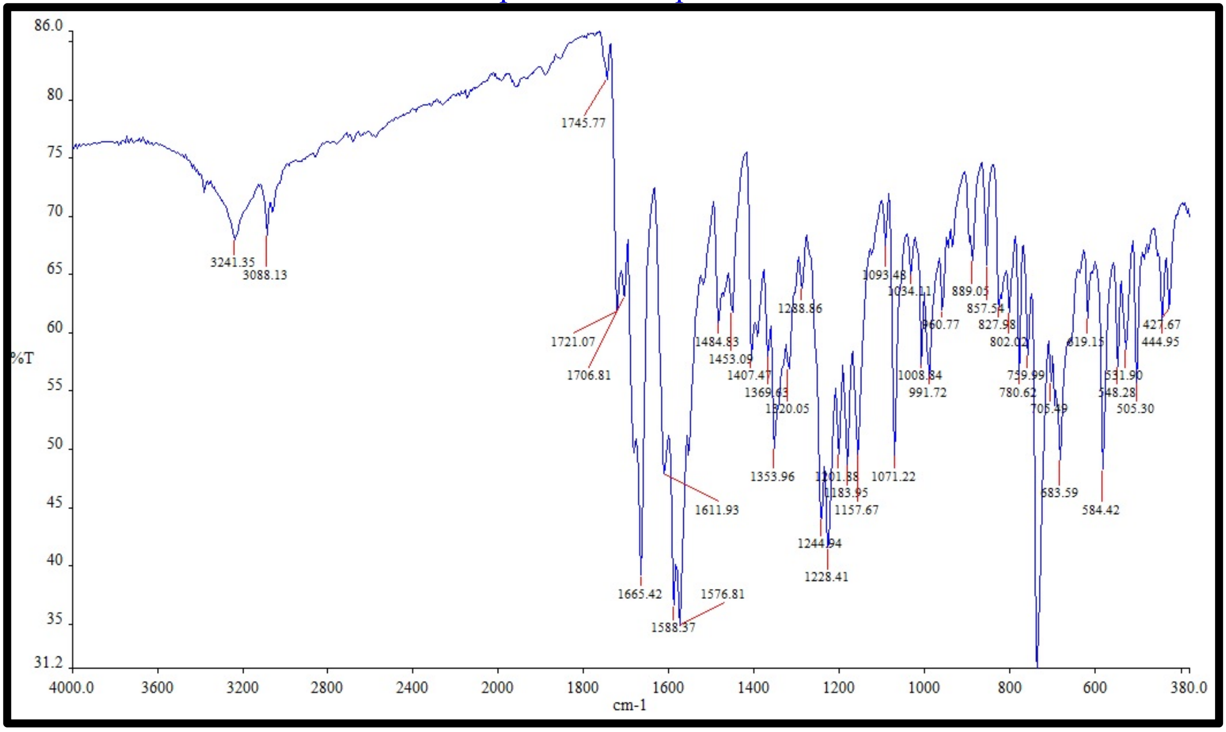

FT-IR spectra of compound **4h**

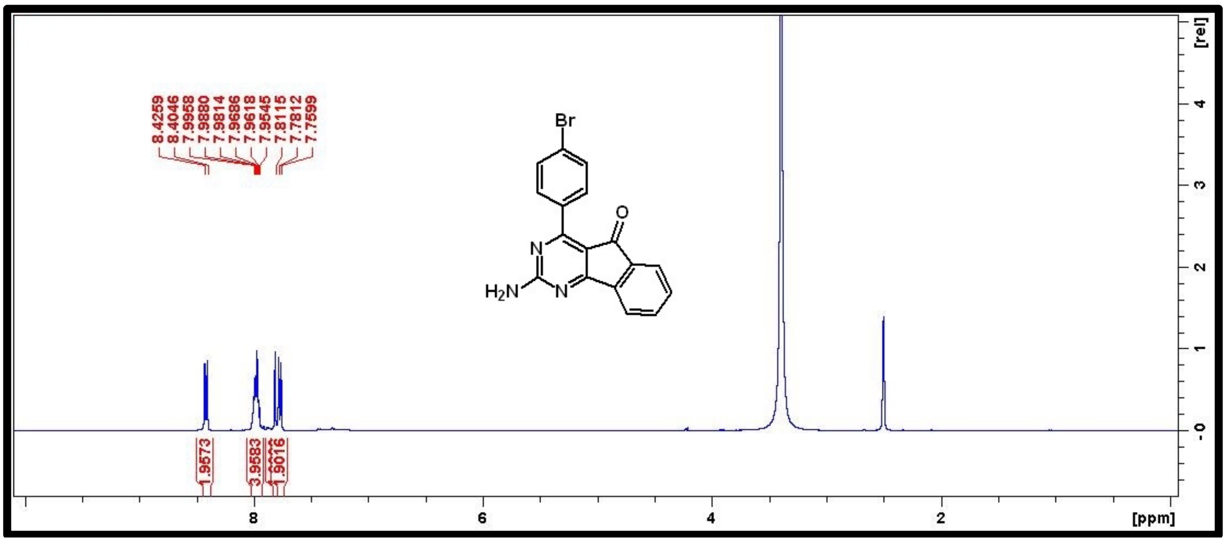

^{1}H NMR spectra of compound **4i**

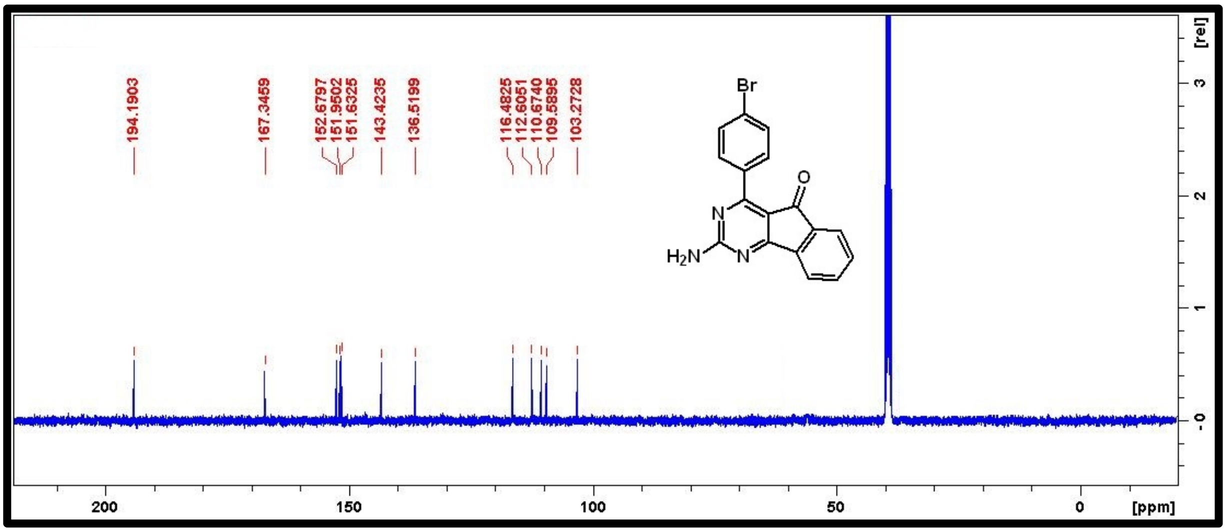

^{13}C NMR spectra of compound **4i**

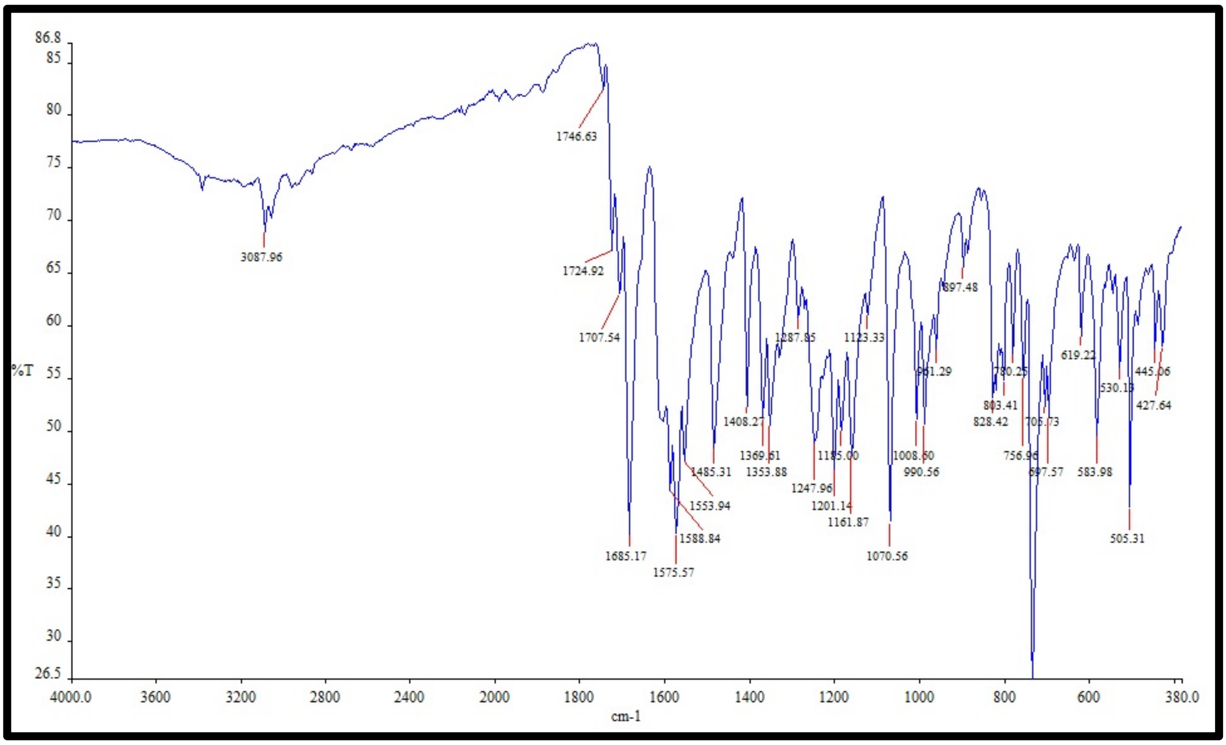

FT-IR spectra of compound **4i**

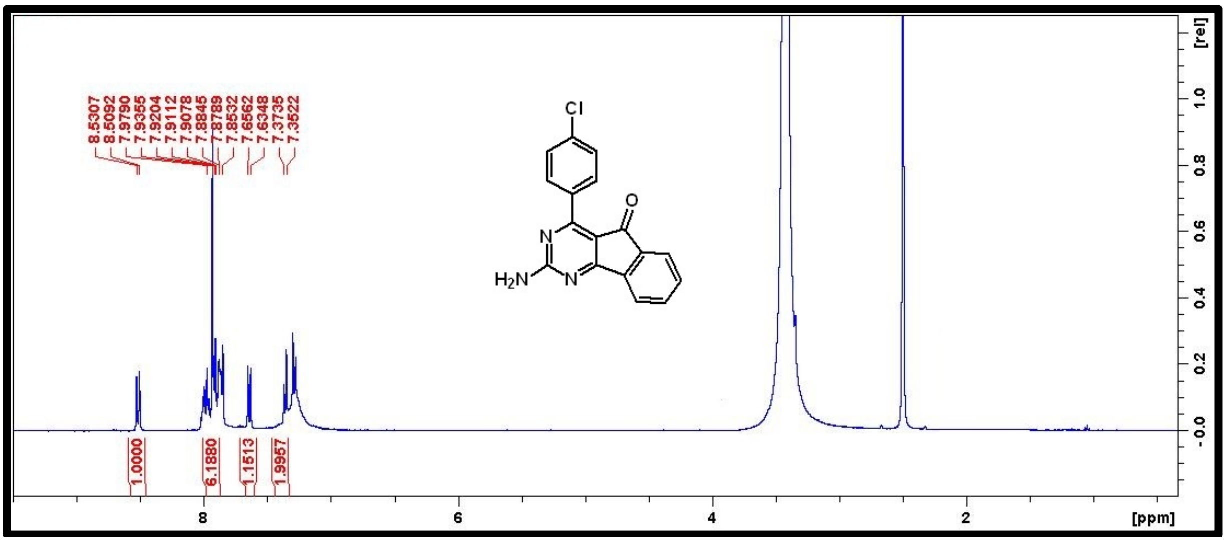

^{1}H NMR spectra of compound **4j**

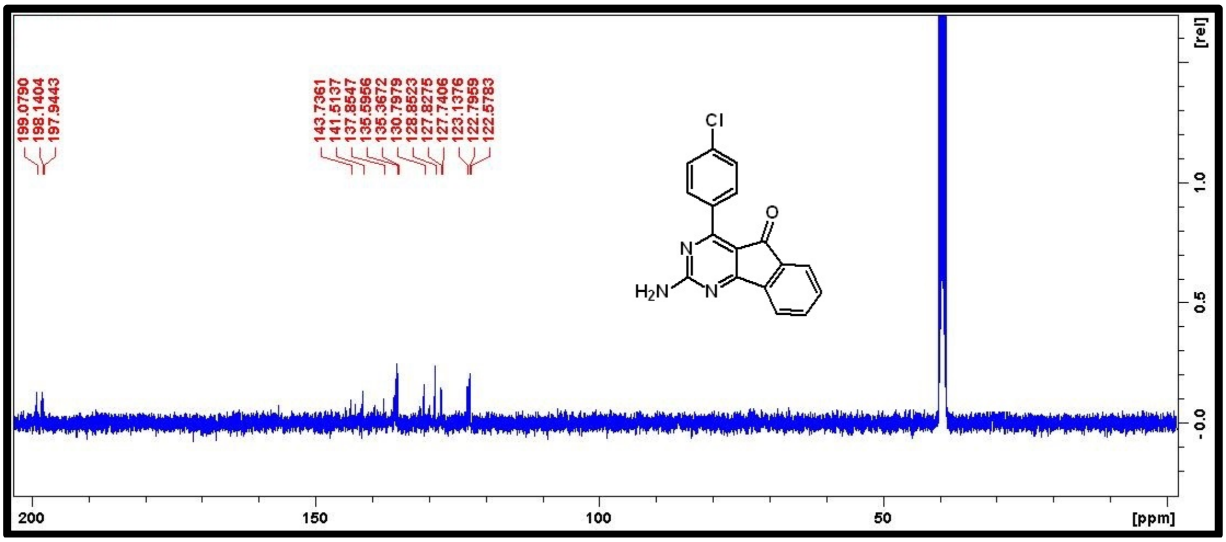

^{13}C NMR spectra of compound **4j**

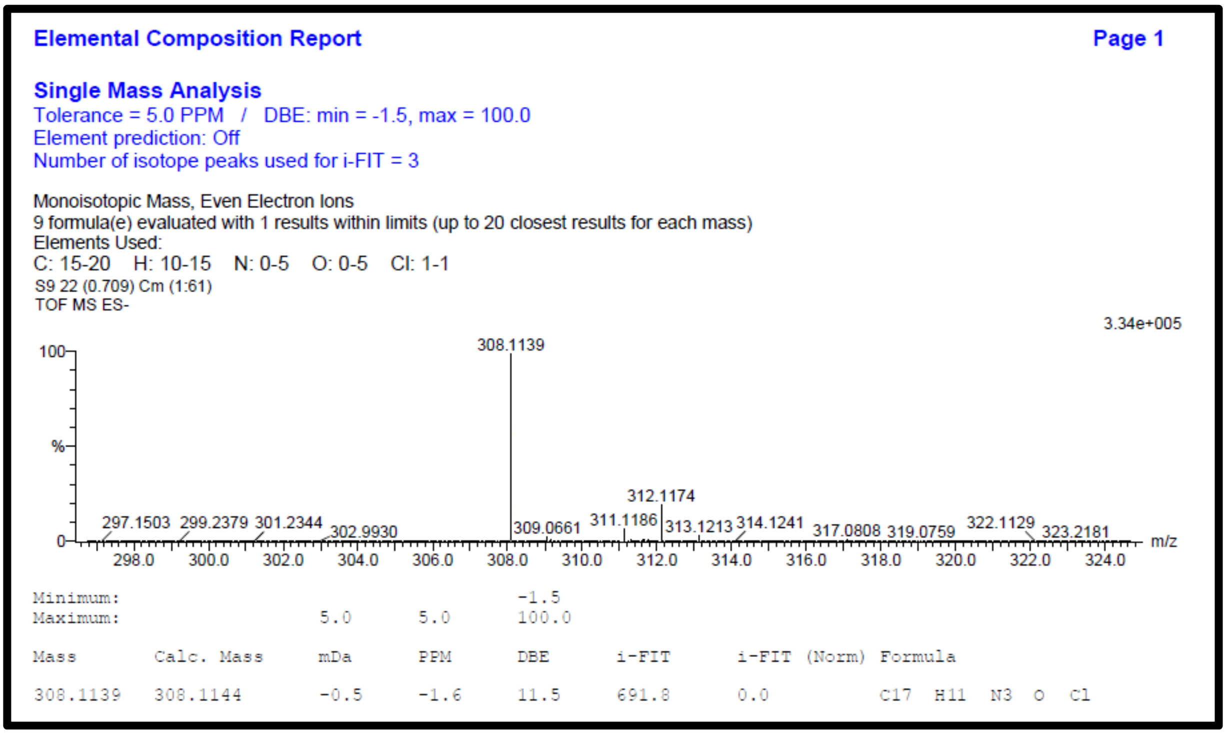

HRMS spectra of compound **4j**

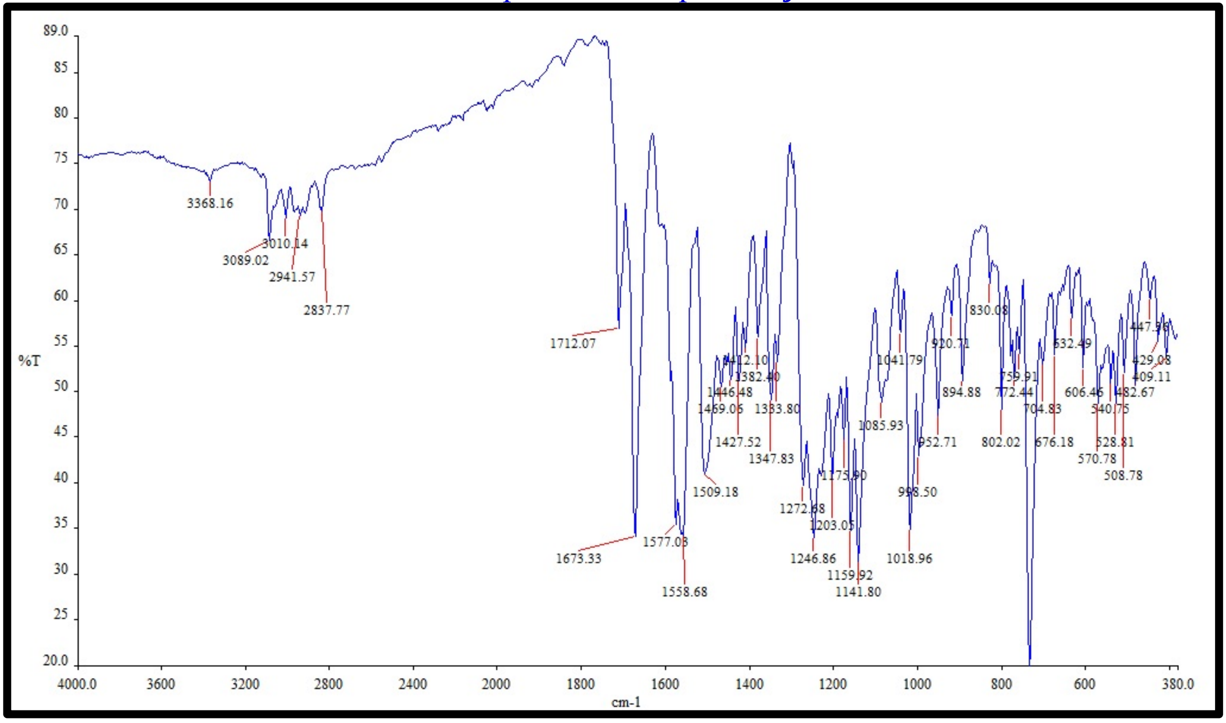

FT-IR spectra of compound **4j**

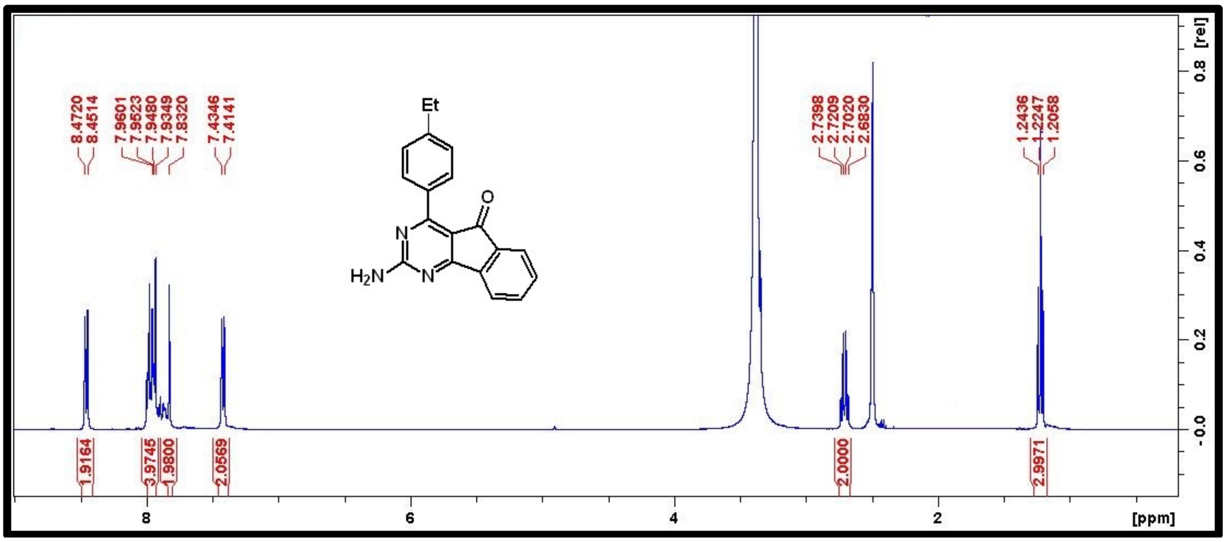

^{1}H NMR spectra of compound **4k**

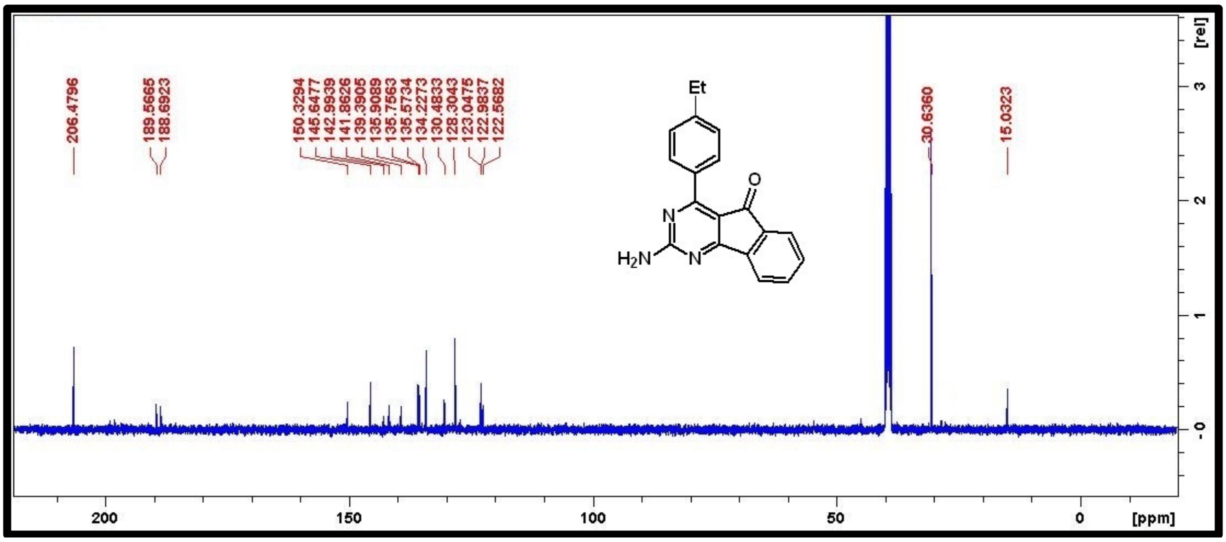

^{13}C NMR spectra of compound **4k**

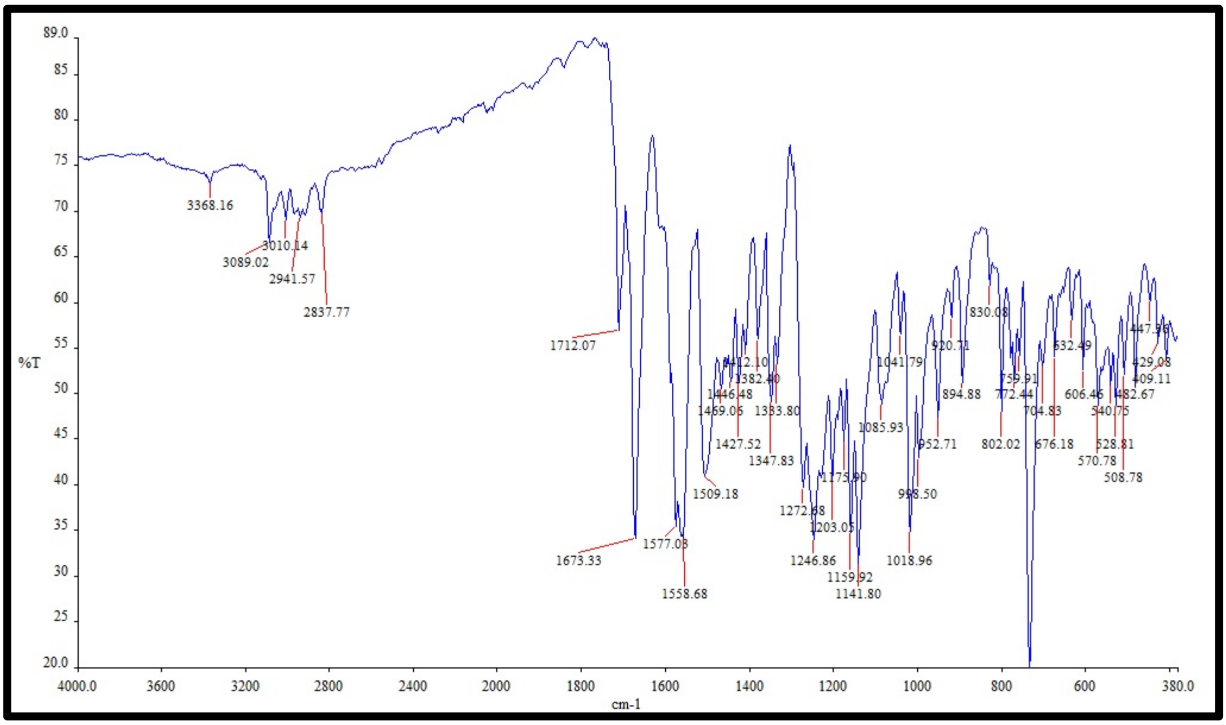

FT-IR spectra of compound **4k**
